# “Dual Disease” TgAD/GSS mice exhibit enhanced Alzheimer’s disease pathology and reveal PrP^C^-dependent secretion of Aβ

**DOI:** 10.1038/s41598-019-44317-w

**Published:** 2019-06-12

**Authors:** Kefeng Qin, Lili Zhao, Crystal Gregory, Ani Solanki, James A. Mastrianni

**Affiliations:** Department of Neurology, The University of Chicago Pritzker School of Medicine, Chicago, USA

**Keywords:** Mechanisms of disease, Alzheimer's disease

## Abstract

To address the question of cross-talk between prion protein (PrP) and Alzheimer’s disease (AD), we generated TgAD/GSS mice that develop amyloid-β (Aβ) plaques of AD and PrP (specifically mutated PrP^A116V^) plaques of Gerstmann-Sträussler-Scheinker disease (GSS) and compared plaque-related features in these mice to AD mice that express normal (TgAD), high (TgAD/HuPrP), or no (TgAD/PrP^−/−^) PrP^C^. In contrast to PrP^C^, PrP^A116V^ weakly co-localized to Aβ plaques, did not co-immunoprecipitate with Aβ, and poorly bound to Aβ in an ELISA-based binding assay. Despite the reduced association of PrP^A116V^ with Aβ, TgAD/GSS and TgAD/HuPrP mice that express comparable levels of PrP^A116V^ and PrP^C^ respectively, displayed similar increases in Aβ plaque burden and steady state levels of Aβ and its precursor APP compared with TgAD mice. Our Tg mouse lines also revealed a predominance of intracellular Aβ plaques in mice lacking PrP^C^ (TgAD/PrP^−/−^, TgAD/GSS) compared with an extracellular predominance in PrP^C^-expressing mice (TgAD, TgAD/HuPrP). Parallel studies in N2aAPPswe cells revealed a direct dependence on PrP^C^ but not PrP^A116V^ for exosome-related secretion of Aβ. Overall, our findings are two-fold; they suggest that PrP expression augments Aβ plaque production, at least in part by an indirect mechanism, perhaps by increasing steady state levels of APP, while they also provide support for a fundamental role of PrP^C^ to bind to and deliver intraneuronal Aβ to exosomes for secretion.

## Introduction

Key histopathological hallmarks of Alzheimer’s disease (AD) are the accumulation of extracellular amyloid-β (Aβ) plaque deposits and intracellular neurofibrillary tangles (NFT) composed of hyperphosphorylated tau^1,2^. However, over the past decade, mounting evidence suggests prion protein (PrP), the central protein responsible for prion disease, may play one or more roles in AD. An early report described an increase in Aβ plaque burden when TgCRND8 mice that express amyloid precursor protein (APP) carrying the Swedish (Swe) and Indiana (Ind) mutations were crossed to the Tg7 mouse that over-expresses wild type (wt) hamster PrP (PrP^C^) at ~13 times normal^3^. However, a subsequent report found no effect on Aβ or APP levels when the PrP gene (*Prnp*) was deleted from an AD mouse model that expresses APPswe and the exon 9 deletion of presenilin-1 (PS-1ΔE9), although they did find that synaptic degeneration was reduced and spatial memory was better preserved compared with the parental line^4^, suggesting a disease-enhancing effect of PrP^C^. Additional roles for PrP^C^ in AD include a suppressor of β-site APP cleaving enzyme (BACE1)^5^ and a receptor for soluble Aβ oligomers that inhibits long term potentiation (LTP)^6^. The latter function has been challenged by several groups^7–9^, although the group that initially proposed PrP^C^ as the receptor for Aβ and mediator of LTP inhibition and neuronal toxicity extended their findings to support PrP^C^ as the signaling mediator for Fyn kinase^10^.

Evidence that AD-related genes influence prion disease (PrD) also exists. In mice with an ablated APP gene, a 13% delay in onset of PrD following inoculation of mouse-adapted sheep scrapie was reported^11^, suggesting APP, or a fragment of it, might promote PrD. Another group found enhanced PrD-related pathology in an AD mouse model inoculated with scrapie compared with scrapie-infected non-Tg mice^12^. These, and several additional reports that show murine and human PrP can bind to Aβ peptides^13–15^ have suggested cross-seeding between PrP and Aβ as the underlying mechanism for this mutual enhancement of disease. In a recent histopathological analysis of AD brains, co-occurrence of AD and PrD lend support to the notion that PrD might promote AD-like pathology, but that analysis concluded that AD does not promote PrD pathology^16^. Based on these conflicting studies we chose to re-examine the inter-relationship between AD and PrD by generating a Tg mouse that simultaneously develops extracellular deposits of Aβ and PrP amyloid. Although others have focused on the potential of mouse-passaged sheep scrapie to enhance AD, extracellular PrP plaques are not a predominant feature of scrapie. We considered that Gerstmann-Sträussler-Scheinker disease (GSS), a PrD with prominent extracellular PrP amyloid deposits, would be better suited to test the potential for heterologous cross-seeding of amyloid between these two diseases. To accomplish this, TgGSS mice that express mouse PrP^A116V^, the mouse homolog of human PrP^A117V^ known to cause GSS^17^, were crossed with a previously established mouse model of AD that co-expresses human APPswe and PS-1ΔE9 transgenes^18^. Our studies with these mice provide two distinct findings. First, we provide additional evidence that cross-talk occurs between PrD and AD, although our results question cross-seeding as the principal mechanism by which PrP augments Aβ plaque production. Second, our studies reveal an important new function of PrP^C^ to facilitate secretion of intraneuronal Aβ via exosomes.

## Materials and Methods

### Transgenic (Tg) mouse lines

The ceAPPswe/PS-1ΔE9 transgenic mouse line^18^ (designated here as TgAD) was obtained from The Jackson Laboratory (Bar Harbor, ME). In this line, the co-integrated APPswe and PS1ΔE9 transgenes are driven by the mouse prion protein (PrP) promoter. The APPswe transgene encodes a chimeric mouse-human APP695 harboring a human Aβ domain and mutations (K595N, M596L) linked to Swedish FAD pedigrees (APPswe), and human presenilin 1 (PS1) lacking exon 9 (PS1ΔE9)that is linked to familial AD^19,20^. TgAD mice (C3HB6 background) were crossed to Tg*Prnp*^−/−^ mice (FVB background)^21^, a gift from Stanley Prusiner, M.D. (UCSF, San Francisco, CA), for more than 8 generations, to establish the TgAD/PrP^−/−^ mouse line on the FVB background. Tg(PrP-A116V) mice (referred to here as TgGSS mice) that model GSS were generated and described by us previously^17^. In short, the transgene incorporates the mouse PrP promoter to express mouse PrP carrying the A116V mutation allelic with 128 V to replicate the homologous PrP^A117V/129V^ genotype linked to human GSS^22^. These mice were originally constructed in Tg*Prnp*^−/−^ mice. TgAD/PrP^−/−^ FVB mice were crossed to TgGSS mice to establish the TgAD/GSS line, which expresses only PrP^A116V^ and the AD-related genes but lacks endogenous PrP^C^. To ensure consistency, littermate controls and only female mice were used. An additional mouse line was created by crossing TgAD/PrP^−/−^ mice to TgHuPrP mice^23^ that express human PrP^C^ on the FVB background at 8x the normal level. As such, TgAD/HuPrP mice express the AD transgene in addition to human PrP^C^ at ~4 times normal, a level that closely compares with that of PrP^A116V^ in TgAD/GSS mice.

### Clinical assessment of mice

TgGSS and TgAD/GSS mice were monitored for signs of GSS, which include ataxia, roughened fur, hunched posture, and impaired righting reflex. We previously described a clinical scoring system that defines 6 stages of disease in our mice^24^ A0 = no ataxia; A1 = a subtle change in gait (wider, lower to the ground), but not definitive ataxia; A2 = persistent and obvious wobble (this stage defines the clear onset of ataxia); A3 = stumble/loss of footing occasionally (once or twice during a 2 min observation); A4 = stumble/loss of footing every few steps; A5 = falling, weight loss generally obvious, clear change in coat roughness, near terminal; A6 = lethargic, very hunched, emaciated. Mice are generally killed during late stage A5 or early stage A6 (A5/A6) when they can no longer feed and death is imminent. Kaplan-Meier survival analysis was used to analyze survival curves and log rank (X^2^) was calculated.

### Tissue processing of mouse brains

Mice were anesthetized with a 2:1 mixture of ketamine (10 mg/ml) and xylazine (2 mg/ml) and slowly perfused *via* cardiac puncture with 20 ml of phosphate-buffered saline (PBS). Isolated brains were bisected longitudinally and the left hemispheres were separated and frozen on dry ice and stored at −80 °C for protein assays. The right hemispheres were stored in 4% paraformaldehyde for 48 h and then transferred to PBS containing 0.05% sodium azide (Sigma-Aldrich, St. Louis, MO) until embedded in paraffin blocks. Sections were cut 5 μm thick and stained with hematoxylin and eosin (H&E) or immunostained.

### Antibodies

For Western blotting, co-immunoprecipitation, and immuofluorescence staining, the following antibodies were used: SAF-32 anti-PrP mouse monoclonal antibody (mAb) (SPI Bio, CA), human Fab anti-PrP D13 antibody (Prionics, CA), anti-APP mouse mAb 22C11 (EMD Millipore, MA), anti-PS1 antibody (ab38323) (Abcam, Cambridge, MA), anti-α-tubulin IgM mouse mAb (Santa Cruz Biotechnology, CA), anti-Aβ42 rabbit antibody PA3-16761 or 44–344 (Thermo Fisher Scientific, Waltham, MA), anti-Aβ_42_-selective mouse mAb MOAB-2 (Abcam), mAb Y188 (Abcam) and 6E10 mouse mAb to detect APP and Aβ (BioLegend, CA), mouse anti-NeuN mAb (Millipore) to label neuronal cell bodies, M78 rabbit mAb to detect intracellular Aβ fibrils^25^ (Pensalfini, Albay *et al*. 2014) (a gift from Gopal Thinakaran, U. Chicago), anti-Cathepsin D (CTSD) rabbit mAb (Abcam), anti-CD63 mouse mAb (Abcam, MA), anti-Alix mouse mAb 3A9 (Cell Signaling), anti-Flotillin-1 mouse antibody (BD Biosciences, CA). Secondary antibodies used in Western blotting included mouse IgG kappa binding protein (m-IgGκ BP) conjugated to horseradish peroxidase (HRP), mouse anti-rabbit IgG-HRP and goat anti-mouse IgM-HRP (Santa Cruz Biotechnology, Dallas, TX). Secondary antibodies for immunofluorescence included Alexa Fluor 647-conjugated AffiniPure goat anti-mouse IgG, rabbit IgG or human IgG (Jackson ImmunoResearch Lab, West Grove, PA), and Alexa Fluor 488-conjugated goat anti-mouse IgG, rabbit IgG or human IgG (Life Technologies, Grand Island, NY).

### Protein analyses

Brain homogenates were prepared in lysis buffer (20 mM Tris-HCl, pH 8.0, 150 mM NaCl, 1 mM EDTA, 0.5% Triton X-100, 0.5% Na-deoxycholate) on ice and stored at −80 °C. Cells were lysed on ice for 30 min in lysis buffer containing a protease inhibitor cocktail (Roche, Mannheim, Germany). After centrifugation at 1,000 rpm for 10 min, the supernatant was collected. A total of 30 μg of protein was separated on a 12.5% tris-glycine SDS-PAGE in loading buffer (0.2 M tris-HCl, pH 6.8, 8% SDS, 40% glycerol, 0.08% bromophenol blue) with β-mercaptoethanol (β-ME). To detect Aβ_42_ peptides, 80 μg of protein from brain homogenates was loaded onto a discontinuous 6% and 16.5% tricine-tris SDS-PAGE in loading buffer without β-mercaptoethanol (β-ME). Fractionated proteins and peptides were transferred to PVDF membranes, washed in Tris-buffered saline with 0.1% Tween 20 (TBST), then boiled for 30 sec and immunoblotted with mouse mAb SAF-32 (1:1,000) for PrP, mAb Y188 (1:10,000) for human and mouse APP, mAb 22C11 (1:5,000) for APP, rabbit Aβ antibody PA3-16761 (1:1,000), rabbit mAb to presenilin 1 (PS1) (1:1,000), mouse mAb to flotillin (1:1,000), rabbit mAb to cathepsin D (CTSD) (1:1,000), and mouse IgM mAb to α-tubulin (1:10,000). In some cases, PVDF membranes were stripped with Restore Western Blot Stripping Buffer (Thermo) and re-probed with different antibodies.

### Immunofluorescence and thioflavin S staining

Brain sections were deparaffinized with xylene (Thermo Fisher Scientific), blocked with 2% bovine serum albumin (BSA) for 1 h, then incubated with rabbit anti-Aβ_42_ antibody PA3-16761 (1:200), mouse anti-Aβ_42_ antibody MOAB-2, mouse mAb SAF-32 (1:100), or mouse anti-NeuN mAb (1:100), overnight. Unbound primary antibodies were removed by a PBS rinse, followed by incubation with secondary antibody, Alexa Fluor 488 F(ab’) fragment of goat anti-rabbit IgG (H + L) (Invitrogen) (1:50) and DyLight 649-conjugated Affini-Pure goat anti-mouse IgG (Jackson ImmunoResearch, Lab, Inc.) (1:50) at room temperature for 1 h, and washed with PBS. To detect intracellular Aβ, brain sections were stained overnight with rabbit mAb M78 (1:100) that recognizes a discontinuous Aβ epitope in Aβ fibrils and mouse mAb 6E10 (1:100) that recognizes APP and Aβ, followed by Alexa Fluor 488 F(ab’) fragment of goat anti-mouse IgG (H + L) (Invitrogen) (1:50) and DyLight 649-conjugated Affini-Pure goat anti-rabbit IgG (Jackson ImmunoResearch, Lab, Inc.) (1:50) secondary antibodies. To visualize amyloid, sections were stained with 1% Thioflavin S (Sigma-aldrich, St, Louis, MO) for 5 min and washed with 70% ethanol and water. Nuclei were stained by incubating with 10 μg/ml 4′-6-diamidino-2- phenylindole (DAPI) (Sigma-Aldrich) at room temperature for 1 min. Sections were washed with PBS, air dried, mounted with Vectashield mounting medium for fluorescence H-1000 (Vector Laboratories, Inc., Burlingame, CA) then observed using a Marianas Yokogawa type spinning disk inverted confocal fluorescence microscope (Zeiss, Germany) and collected images were montaged using Slidebook 5.5 software.

### Histopathological assessments

Plaque burden was calculated as the area stained by Thioflavin S relative to the total brain section area, or the area of antibody staining for Aβ_42_ or PrP, relative to the entire brain section area, using an NIH ImageJ plug in and reported as the mean area ± S.D. In most cases, this data was normalized to TgAD mice unless otherwise specified. Each brain section was fully scanned by automated and systematic imaging of individual overlapping 20X fields that were montaged together to produce full section images for quantitation of amyloid plaques. Spongiform degeneration was similarly assessed with NIH ImageJ using 10X fields that were montaged. In general, 3 parasagittal sections that cut through cortex, hippocampus, and cerebellar regions were obtained from each mouse brain and 6 brains were assessed per group, unless otherwise stated. For analysis of plaque counts differentially labeled by M78 and 6E10, total counts were determined by manually counting plaques co-labeled (intracellular) and those labeled only by 6E10 (extracellular) and summing the two. Plaque counts were determined as the mean ± S.D. of plaques within each parasagittal brain section, unless otherwise stated.

### Cell culture

N2a cells stably expressing the APP Swedish mutation (N2a-APPswe) were cultured in Dulbecco’s modified Eagle’s medium (DMEM) (Invitogen, Carlsbad, CA) supplemented with 10% fetal bovine serum (FBS) at 37°C and 5% CO_2_. To transiently knock down expression of the endogenous mouse *Prnp* gene without affecting recombinantly-expressed PrP, two siRNAs that target sequences within the 3′ untranslated region (3′-UTR) of mouse *Prnp* were used: Prnp3, targeting sequence CCC TAT GTT TCT GTA CTT CTA, and Prnp4, targeting sequence CTG ATT GAA GGC AAC AGG AAA (Qiagen, Valencia, CA). A non-interfering siRNA (Qiagen) was used as control. Cells were transfected with siRNA (20 nM) using RNAiMAX reagent per the manufacturer’s instructions (Invitrogen). In some cases, cells were co-transfected with siRNA (Prnp3 and Prnp4) and the pCB6 expression vector carrying WT PrP or PrP^A116V^, using Lipofectamine 2000 (Invitrogen). After 24 h of incubation, the transfection reagent containing siRNA and/or expression vectors was removed and replaced by OPTI-MEM I (Invitrogen) for 24 h. Media was collected 48 h post-transfection and stored at −20°C for subsequent analysis.

### Immunofluorescence cell staining

Cells were cultured on coverslips and transfected for 48 h, washed with PBS, fixed with 4% formaldehyde in PBS at room temperature for 15 min, washed with PBS, followed by incubation with 0.1% Triton X-100 in PBS for 5 min, to permeablize membranes. Cells were blocked with 2% BSA in PBS for 1 h, then incubated overnight at room temperature, with mouse anti-PrP mAb SAF-32 (1:200) and rabbit anti-Aβ_42_ antibody 16761 (1:200). Following a wash, cells were incubated with Alexa Fluor 488 goat anti-mouse IgG (Invitrogen) (1:50), and DyLight 649-conjugated Affini-Pure goat anti-rabbit IgG (Jackson ImmunoResearch Labs) (1:50) at room temperature for 1 h, then washed with PBS. To stain nuclei, cells were incubated with 10 μg/ml DAPI (Sigma-Aldrich) for 1 min. Cells were given a final wash with PBS, air-dried, then mounted and visualized using a Marianas Yokogawa type spinning disk inverted confocal fluorescence microscope.

### Sandwich enzyme-linked immunosorbent assay (ELISA)

#### Aβ measurements

Mouse anti-Aβ mAb 6E10 (BioLegend) (1:400) in carbonate buffer (CB) was used to coat a 96-well microplate at 4 °C overnight. Between steps, the plate was washed with phosphate buffered saline containing 1% Tween 20 (PBST). The wells were blocked with PBST containing 1% BSA (BSA-PBST) at room temperature for 1 h. Serial dilutions of synthesized Aβ_42_ (GenScript, USA) in BSA-PBST were added to the wells to calculate the standard curve. Ten percent brain homogenates from wild type (WT) FVB, TgPrP^−/−^, TgGSS and TgHuPrP mice prepared in RIPA buffer containing the soluble Aβ were used as RIPA-extracted samples. Brain homogenates were mixed with 70% formic acid (FA) and homogenized again following centrifugation in a TLA 100.3 rotor at 45,000 rpm for 1 h. The supernatant was diluted with FA neutralization buffer (1:20) and then used as FA-extracted samples. RIPA- or FA-samples were serially diluted in BSA-PBST and added in wells of the plate for incubation at 4 °C overnight. Wells of the plate were then incubated with Aβ42 polyclonal antibody (Invitrogen 44–344) (1:400) followed by anti-rabbit IgG-HRP (Santa Cruz) (1:2,500) at room temperature for 1 h. After incubation with the developing solution, 3,3″5,5″-tetramethylbenzidine (TMB) at 37 °C for 5–20 min, 2N HCl was added to stop the reaction. O.D. at 450 nm was measured for calculating Aβ concentrations.

#### PrP measurements

Human anti-PrP antibody D13 (1:400), which recognizes mouse PrP, was dissolved in CB and used to coat the wells of a 96-well microplate at 4 °C overnight. Between all steps, the plate was washed with PBST. Wells were blocked with BSA-PBST at room temperature for 1 h. Serial dilutions of recombinant PrP (Invitrogen) in BSA-PBST were added to the wells to generate a standard curve. The 10% brain homogenates from WT (FVB), TgPrP^−/−^, TgGSS and TgHuPrP mice were serially diluted in BSA-PBST and added to wells of the plate and incubated overnight at 4 °C. Mouse anti-PrP mAb SAF-32 (SPI bio) (1:400) was then added, followed by anti-mouse IgG BP-HRP (Santa Cruz) (1:2,500) at room temperature for 1 h. After incubation with TMB at 37 °C for 5–20 min, 2N HCl was added to stop the reaction and read on a plate reader at O.D. 450 nm to calculate PrP concentrations.

### Aβ and PrP binding studies

The synthesized human Aβ42 peptide (GenScript, NJ) (1,400 nM) was dissolved in CB (50 µl) and added to wells of a 96-well microplate and incubated at 4 °C overnight. The plate was washed with PBST between each step. The wells were blocked with BSA-PBST at room temperature for 1 h. Serial dilutions of recombinant human PrP (Invitrogen) dissolved in BSA-PBST were added to the wells to generate a standard curve. The 10% brain homogenates from WT FVB, TgPrP^−/−^, TgGSS, and TgHuPrP mice were serially diluted in BSA-PBST, added to wells and incubated at 4 °C overnight. Wells were then incubated with mouse anti-PrP mAb SAF-32 (SPI bio) (1:400) followed by the anti-mouse IgG BP-HRP (Santa Cruz) (1:2,500) at room temperature for 1 h. After incubation with TMB at 37 °C for 5–20 min, 2N HCl was added to stop the reaction. O.D. at 450 nm was measured to calculate the binding.

### ELISA measurement of Aβ42 in N2a-APPswe cells

N2a-APPswe cells were grown in 24 well cell culture plates for 24 h to nearly 100% confluence, then cells in each of 6 wells were recovered with 500 µl of OPTI-MEM media (PrP^+^) or transfected with siRNA (Prnp3 and Prnp4) to knock down endogenous PrP^C^ expression (PrP^−^), or co-transfected with siRNA against PrP^C^ and plasmid pCB6-PrP^A116V^ for 24 h. Media (500 µl) was collected and cells were lysed with 100 µl of cell lysis buffer. A solid-phase sandwich ELISA kit for detection of human Aβ_42_ (Invitrogen) was used to quantify the total Aβ_42_ in cell lysates and cell media. Human Aβ standard was serial diluted to generate a standard curve. The cell lysate and media were diluted for Aβ_42_ measurement following the manufacturer’s instructions. The final amount of Aβ was calculated as pg of total intracellular or media Aβ.

### Co-immuoprecipitation (Co-IP)

Brain homogenates prepared from cortex of mice or lysates of N2a-APPswe cells were diluted (1:1) with binding buffer, 0.05 M sodium borate, pH 8.0, 0.15 M NaCl, and then incubated with 10 μl of Protein G-Sepharose beads (Biovision, Mountain View, CA) (pre-washed with the binding buffer) with gentle agitation at 4 °C for 1 h to reduce non-specific binding. After centrifugation at 10,000 × *g* for 10 min, the supernatants were used for immunoprecipitation (IP). Samples were incubated at room temperature for 1 h with the IP-antibodies, mouse anti-PrP mAb SAF-32 at a ratio of 1:100 (v/v). After addition of 10 μl pre-washed Protein G-Sepharose beads, the mixture was rolled at 4 °C overnight. Antigen-antibody-Protein G-sepharose complexes were collected by centrifugation at 14,000 r.p.m. for 10 min and washed 3 times with 1 ml of the binding buffer. Proteins were eluted with 60 μl of elution buffer, 0.1 M citric acid, pH 2.75. The eluate was mixed with 20 μl of 4 × SDS-loading buffer without β-ME and adjusted to pH 8.0 with 1 μl of 10 M NaOH, and then boiled for 5 min. Samples (30 μl each) were separated using 12.5% SDS-PAGE to detect PrP or 6% and 16.5% discontinuous tricine-tris SDS-PAGE gels for Aβ detection. Following transfer to PVDF membranes, proteins and peptides were probed with human anti-PrP antibody D13 (1:1000) or rabbit anti-Aβ_42_ antibody PA3-16761 (1:1000).

### Detection of nuclear and cytoplasmic Aβ

Fresh frozen brain samples (80 mg) from TgAD, TgAD/PrP^−/−^ and TgAD/GSS mice were fractionated with the NE-PER Nuclear and Cytoplasmic Extraction Reagents kit (Thermo Fisher Scientific) according to the manufacturer’s protocol. Aβ was separated and detected by Western blot using a discontinuous 6% and 16.5% tricine-tris gel.

### Exosome preparation

N2a-APPswe cells were transfected with a non-silencing control siRNA or active siRNA against *Prnp* (Prnp3 plus Prnp4) and/or transfected with a pCB6 expression vector containing either PrP^A116V^ or WT mouse PrP. After incubation for 24 h, siRNA and the transfection reagent were removed and replaced by OPTI-MEM I (Invitrogen) for 24 h incubation. Exosomes were prepared using ExoQuick-TC ULTRA EV Isolation Kit for Tissue Culture Media (System Biosciences, SBI, Palo Alto, CA), following the manufacturer’s instructions^26,27^. Briefly, cell culture media was collected and centrifuged at 3,000 × g for 15 min to remove debris. The supernatant was mixed with ExoQuick-TC and incubated at 4 °C overnight. The mixture was centrifuged at 3,000 × g for 10 min. The pellet was resuspended in Buffer A and passed through the purification column. The purified exosomes were then prepared for transmission electron microscopy (TEM) by negative staining, and for Western blotting to assess Aβ, PrP, APP, and exosome markers Alix, flotillin, and CD-63 proteins.

### Study approvals

All animal studies and experimental protocols were approved by the Institutional Animal Care and Use Committee and Institutional Biosafety Committee at the University of Chicago. All experiments were performed in accordance with the relevant guidelines and regulations.

### Statistical analysis

An ANOVA test or Student’s *t*-test was applied to assess the relationship between different variables where appropriate. For most analyses, an ANOVA was applied to define a difference among treatment groups, which was followed by a post-hoc multiple comparisons test to define specific group differences, using GraphPad (Prism) software. Values of *p* < 0.05 were considered significant and *p* < 0.01 very significant.

## Results

### TgAD/GSS mice display enhanced clinical disease and histopathological features

To determine if the presence of PrP amyloid promotes Aβ plaque generation “dual-disease” mice were generated by crossing Tg ceAPPswe/PS-1ΔE9 mice that model AD by co-expression of human APP with the Swedish mutation (APPswe) and presenilin-1 (PS-1) carrying the exon 9 deletion (hereafter referred to as TgAD mice) with Tg(PrP-A116V) mice (hereafter referred to as TgGSS mice) that express mouse sequence PrP^A116V^, a homolog of human PrP^A117V^ that causes GSS^22^. The latter mice replicate key clinicopathological features of GSS, including progressive gait ataxia that leads to severe debilitation and death at ~170 days of age and the presence of extracellular PrP amyloid plaques, predominantly within the cerebellum^17,24^. Because TgGSS mice were constructed on a PrP knockout (PrP^−/−^) background, TgAD mice were first crossed with TgPrP^−/−^ mice to eliminate any confounding effect of endogenous PrP^C^. Thus, whereas TgAD mice express endogenous PrP^C^, TgAD/GSS mice express AD transgenes and PrP^A116V^, but not PrP^C^.

**Figure 1:**
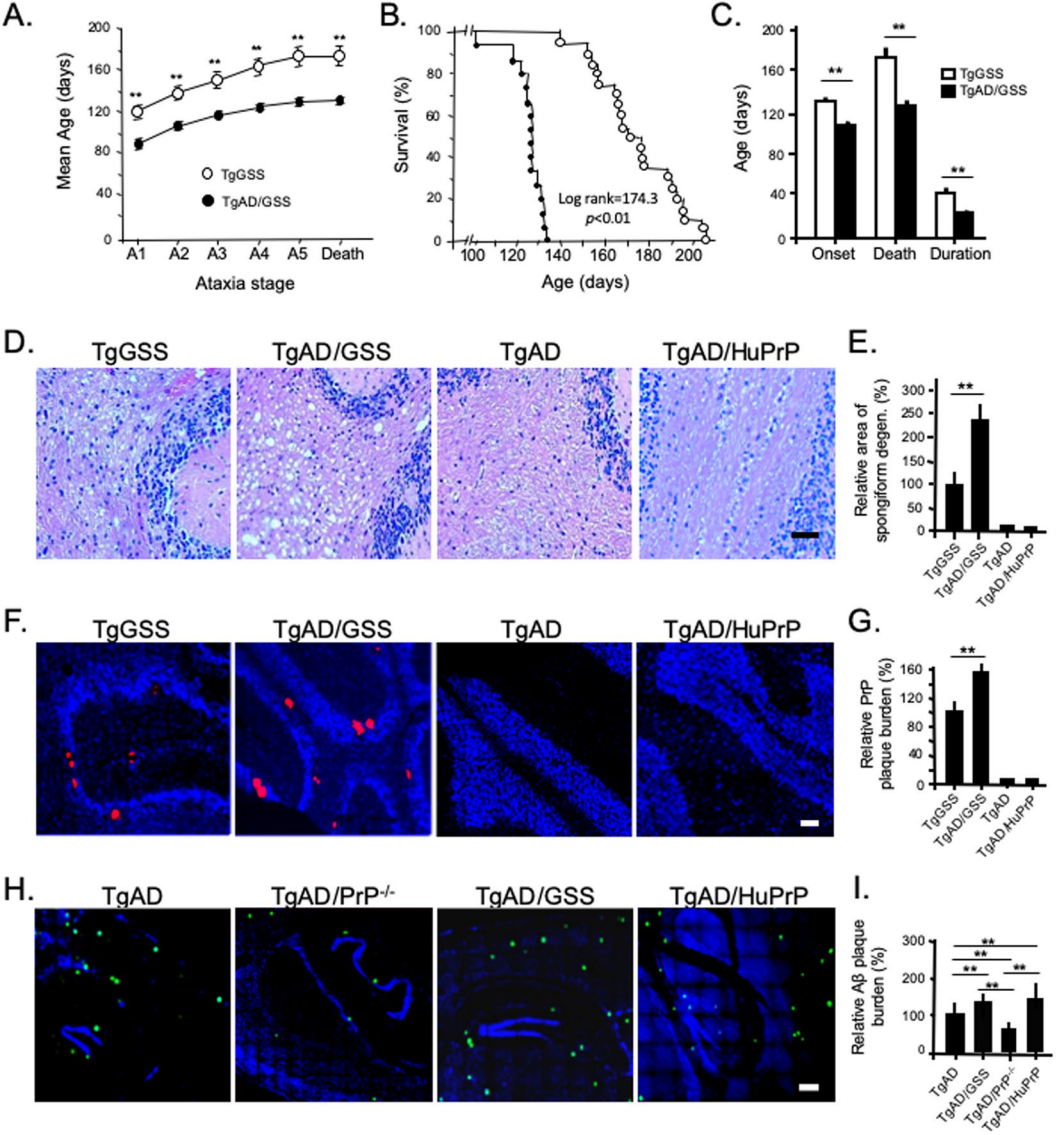
Reduced survival and enhanced AD and PrD pathology in TgAD/GSS mice. (**A**) Mean age at which TgGSS (open circles, n = 20) and TgAD/GSS (closed circles, n = 15) mice reach each clinical stage of ataxia (see Methods). **p < 0.01, Student’s *t*-test applied to each stage. See Supplemental Table 1A for actual values. (**B**) Kaplan-Meier survival curve of TgGSS (open circles) and TgAD/GSS (closed circles) mice. X^2^ (log rank) = 174.3, *p* < 0.01. (**C**) Bar graph comparing age at onset ± S.D in TgGSS (open bars, n = 20) vs TgAD/GSS (closed bars, n = 15) mice (132.1 ± 12.7 vs 106.1 ± 6.0 d), age at death ± S.D. (174.6 ± 18.4 vs 126.0 ± 8.2 d), and disease duration ± S.D. (42.5 ± 10.6 to 21.8 ± 5.8 days) (**p < 0.01), Student’s *t*-test applied to each group. (**D**) Representative H&E stained sections of cerebellum from age-matched (~126 ± 8 day-old, i.e. ~4.2 month-old) mice of each Tg line showing differences in spongiform degeneration within the cerebellum. Magnification 20x. Scale bar = 50 µm. (**E**) Bar graph comparing the relative area of spongiform degeneration from each line displayed in D, normalized to the TgGSS group. Brain sections were prepared as a parasagittal slice that included cortex, hippocampus, and cerebellar structures. The relative area of spongiform degeneration was calculated as a fraction of the total area of brain section using NIH ImageJ (see Methods). **p < 0.01, ANOVA and post hoc multiple comparisons test. Actual values: TgGSS = 100 ± 25.7%, TgAD/GSS = 241.4 ± 45.6%, TgAD = 0, TgAD/HuPrP = 0 (n = 6 mice per group, 3 brain sections per mouse). (**F**) Representative full thickness confocal immunofluorescence images of fixed cerebellar sections from ~4.2 month-old Tg mice from each line labeled with anti-PrP SAF-32 mAb to visualize PrP plaques. Nuclei were stained with DAPI (blue). Sections were 5 μm thick. Original magnification 20x. Scale bar = 100 µm. (**G**) Bar graph comparing the relative plaque burden (%) within each mouse line normalized to TgGSS mice. Area of PrP plaque immunofluorescence was calculated as the fraction of total brain section area, using an NIH ImageJ plugin (see Methods). **p < 0.01, ANOVA and post hoc multiple comparisons test. Actual values: TgGSS = 100 ± 24.5%, TgAD/GSS = 154.1 ± 15.3%, TgAD = 0, TgAD/HuPrP = 0 (n = 6 mice per group, 3 sections per mouse brain). (**H**) Representative confocal immunofluorescence images of mouse brain show Aβ plaques within the cortical/hippocampal region of ~4.2 month-old mouse lines with AD transgenes and differing PrP transgenes. Aβ was detected using rabbit anti-Aβ_42_ antibody (PA3-16761). Original magnification 20x. Scale bar = 100 µm. (**I**) Bar graph comparing Aβ plaque burden in each Tg mouse line. The area of anti-Aβ antibody staining relative to the total area of brain section was determined using NIH ImageJ and plotted as a percentage normalized to TgAD mice. **p < 0.01, ANOVA and post hoc multiple comparisons test, (n = 6 mice per group, 3 sections per mouse brain). See Supplemental Table 1B for actual values.

TgAD/GSS mice (n = 15) displayed a more aggressive clinical phenotype than TgGSS mice (n = 20). Based on a clinical scoring system (see Methods) the onset of ataxia (stage A2) occurred at 106.1 ± 6.0 days (~3.5 months) in TgAD/GSS mice compared with 132.1 ± 12.7 days (~4.4 months) in TgGSS mice (*p* < 0.01) (Fig. 1A,C) and TgAD/GSS mice reached each subsequent clinical stage sooner (Fig. 1A). Death occurred at 126.0 ± 8.2 days (~4.2 months) in TgAD/GSS mice compared with 174.6 ± 18.4 days (~5.8 months) (*p* < 0.01) in TgGSS mice, reducing disease duration from 42.5 ± 10.6 to 21.8 ± 5.8 days (*p* < 0.01) (Fig. 1B,C).

Compared with TgGSS mice, TgAD/GSS mice had more prominent spongiform degeneration (Fig. 1D,E) and PrP plaques (Fig. 1F,G). This was specific to PrP^A116V^ expression, as GSS-related pathology was not observed in age-matched TgAD mice and TgAD/HuPrP mice that over-express wild-type (WT) human PrP^C^ at a comparable level as PrP^A116V^ in TgAD/GSS mice (Fig. 1D–G). Using an NIH ImageJ plug-in, we measured an overall 2.5 fold increase in the area of spongiform degeneration (Fig. 1E) and a 50% increase in the area of PrP plaque burden (Fig. 1G) in end-stage TgAD/GSS compared with age-matched (~4.2 month-old, i.e. ~126 ± 8 days) TgGSS mice.

The above data suggest that GSS-related pathology is augmented by co-expression of AD-related transgenes. We then asked whether AD-related pathology was reciprocally promoted by PrP^A116V^ expression. As reported by others^28^, Aβ plaques were detected at low levels in the cortex and hippocampus of ~4.2 month-old TgAD mice and these were increased in terminally ill (~4.2 month-old) TgAD/GSS mice (Fig. 1H**)**. To assess whether the high level of expression or the amyloidogenic PrP^A116V^ is responsible for the increase in Aβ burden, we compared brain sections from TgAD/HuPrP that express WT PrP^C^ at similar levels as PrP^A116V^ in TgAD/GSS mice in addition to TgAD mice lacking PrP^C^ (i.e TgAD/PrP^−/−^). TgAD/HuPrP mice had higher levels of Aβ plaques than age-matched TgAD mice whereas TgAD/PrP^−/−^ mice had the lowest (Fig. 1H). These differences were quantified using an NIH ImageJ plug in to assess the area of anti-Aβ antibody staining relative to the total area of brain section and normalized to age-matched TgAD mice (Fig. 1l). Compared with TgAD mice (100 ± 9.2%), plaque burden was 130% in TgAD/GSS mice, 136% in TgAD/HuPrP mice, and 42.3% in TgAD/PrP^−/−^ mice (Fig. 1I). These results suggest a direct correlation between PrP expression and Aβ plaque burden. They also suggest that similar expression levels of PrP^C^ and PrP^A116V^ induce similar increases in Aβ burden. Thus, the more amyloidogenic PrP^A116V^ does not appear to induce Aβ plaque production more effectively than PrP^C^.

### Elevated disease-related proteins in TgAD/GSS mice

**Figure 2:**
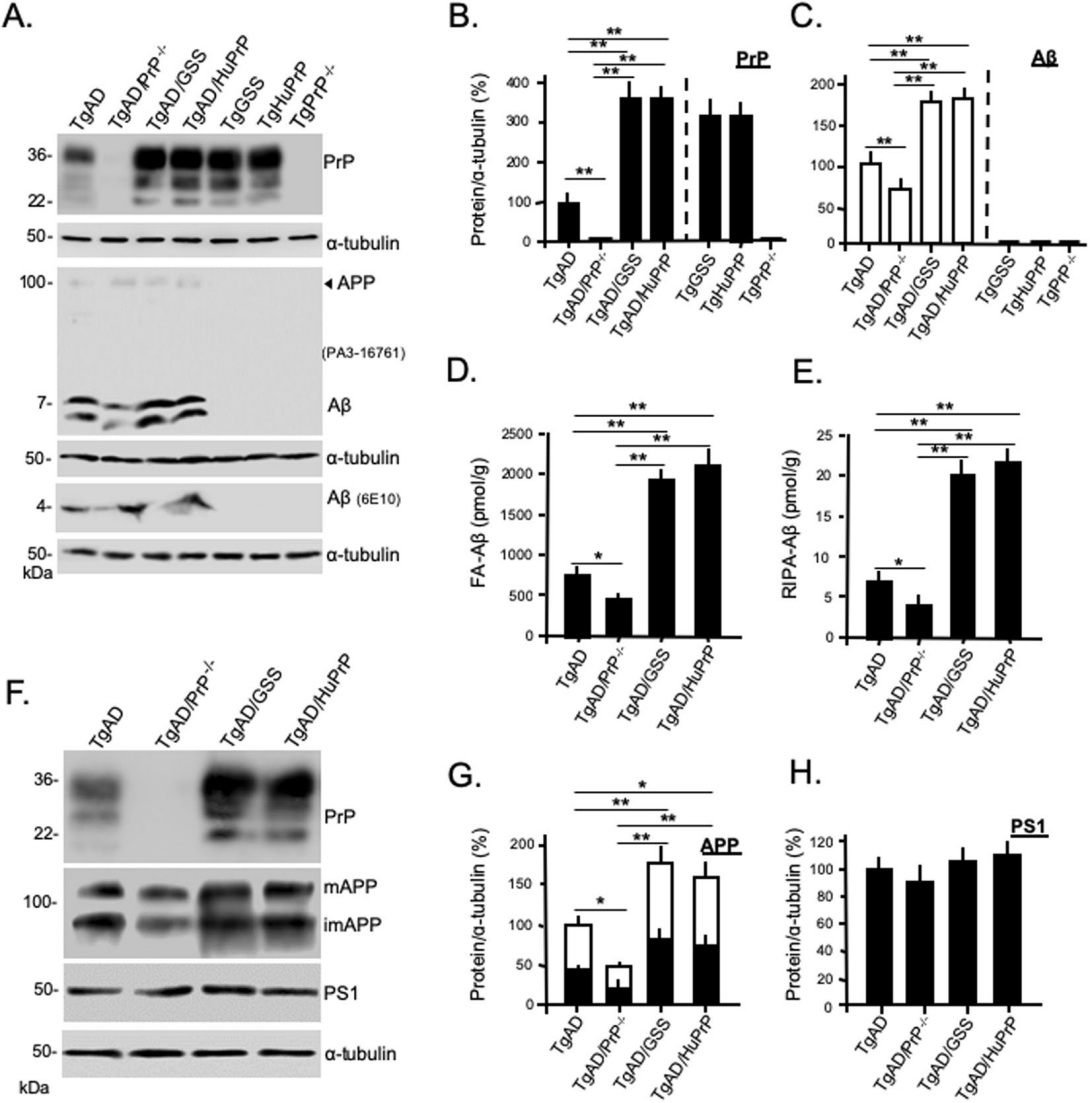
Steady state levels of AD and PrD related proteins in Tg lines. (**A**) Western blots comparing PrP and Aβ steady state levels in each mouse line at ~4.2 months. Freshly harvested brain from mice was homogenized and 30 μg of total protein from each was separated on 12.5% SDS-PAGE to assess PrP and α-tubulin as a loading control. To detect Aβ_42_, 80 μg of total protein was separated on a discontinuous (6%/16.5%) tricine-tris gel. Antibodies used were mAb SAF-32 (PrP) and PA3-16761 or 6E10 (Aβ). Predicted APP position is labeled by arrowhead to demonstrate the selectivity of PA3-16761 for Aβ over APP. Each blot represents 1 of 3 replicates prepared from 3 mice per Tg mouse line. The same samples were run for each of the 3 gels displayed and each gel was re-probed with α-tubulin antibody as a loading control. The densitometric signals of PrP **(B)** and Aβ (PA3-16761) **(C)** were measured using Quantity One software (Bio-Rad) and normalized to signals of α-tubulin. The values were plotted as a relative percentage using age-matched TgAD mice as the reference (i.e. 100%). Bars are means ± S.D. of 3 independent experiments, as in A. See Supplemental Table 2A for actual values. (**D**,**E**) Bar graphs comparing ELISA measurements (see Methods) of Aβ concentrations from brain homogenates of TgAD, TgAD/PrP^−/−^, TgAD/GSS and TgAD/HuPrP mice following extraction with formic acid (**D**) or RIPA buffer (**E**) (n = 3 mouse brains per group, 3 dilutions per experiment, and 2 independent experiments). See Supplemental Table 2B for actual values of FA-Aβ and RIPA-Aβ concentrations. (**F**) Western blots prepared as in A to assess relative levels of PrP (SAF-32), immature APP (imAPP), mature APP (mAPP) (mAb 22 C11), and PS1 (ab38323) in ~4.2 month-old TgAD mice with differing PrP expression levels and/or sequences. Each blot represents 1 of 3 replicates prepared from 3 mice per Tg line. Samples were loaded on one gel to probe for PrP, stripped, then re-probed for APP, PS1 and α-tubulin. (**G**) Bar graph comparing the total densitometric signals of APP, with immAPP (open bar) and mAPP (solid bar) fractions represented within each bar, measured with Quantity One (Bio-Rad) software and normalized to α-tubulin signals. Values were plotted as the relative percent of the corresponding signal in age-matched TgAD mice. Bars are mean ± S.D. of values from blots prepared from 3 mice, as in (**F**). (**H**) Bar graph comparing densitometric signals of steady state PS1, as in (**F**) and expressed as the relative percent of the corresponding signal in age-matched TgAD mice. Bars are mean ± S.D. of values from blots prepared from 3 mice, as in (**F**). See Supplemential Table 2C for actual values. For all above bar graphs, ANOVA and post hoc multiple comparisons tests were performed on each, and bars with asterisks represent differences between the specific groups *p < 0.05, **p < 0.01.

Based on the increased plaque pathology, we predicted that steady state levels of PrP and Aβ would be increased in TgAD/GSS and TgAD/HuPrP mice. Western blots were prepared from brain homogenates of age-matched (~4.2 month-old) mice from each Tg mouse line, followed by semi-quantitative densitometry **(**Fig. 2A–C). Compared with TgGSS, TgAD/GSS mice had slightly but significantly higher steady state levels of PrP^A116V^, consistent with the increase in PrP plaque burden (Fig. 2B). In agreement with the relative changes in Aβ plaque burden, an increase in steady state PrP^C^ relative to control TgHuPrP mice was also evident in TgAD/HuPrP mice (Fig. 2B). Aβ levels were increased in TgAD/GSS and TgAD/HuPrP mice and reduced in TgAD/PrP^−/−^ mice compared to TgAD mice (Fig. 2C). To ensure the Western blot measures did not reflect selectivity for soluble Aβ we used ELISA to measure and compare formic acid-extracted insoluble Aβ (FA-Aβ) (Fig. 2D) and RIPA-extracted soluble Aβ (RIPA-Aβ) (Fig. 2E) in each mouse line. Although the concentration of FA-Aβ was ~100 fold higher than RIPA-Aβ, the proportionate changes in Aβ relative to the levels of PrP were similar to RIPA-extracted Aβ and the Western blot assessment.

We next asked whether the changes in Aβ levels reflected changes in either amyloid precursor protein (APP), the precursor for Aβ, or PS1, the other AD-related transgene in TgAD mice and the principal component of gamma-secretase that cleaves APP to produce Aβ. Surprisingly, we found increases in steady state levels of immature (i) (N-glycosylated) and mature (m) (N-glycosylated, O-glycosylated, and tyrosol-sulfated) forms of APP in TgAD/GSS and TgAD/HuPrP mice in addition to a reduction of APP in TgAD/PrP^−/−^ mice, compared with TgAD mice (Fig. 2F,G). In contrast, PS1 levels did not differ among the Tg mouse lines (Fig. 2F,H).

### Reduced association of PrP^A116V^ with Aβ

**Figure 3:**
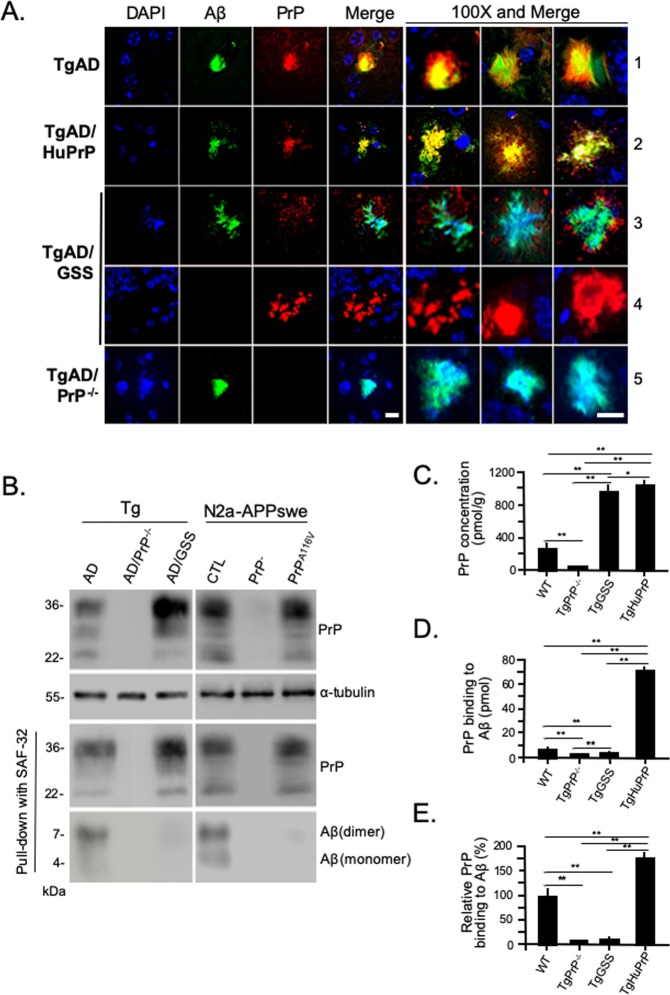
Impaired interaction between PrP^A116V^ and Aβ. (**A**) Representative confocal immunofluorescence images of plaque deposits from each mouse line at ~4.2 months, immunostained for Aβ_42_ and PrP. Nuclei were stained with DAPI. Separate channels are displayed, along with merged images of representative plaques from each line. The merged image and two additional representative merged images from each line are displayed at 100X magnification. PrP^C^ colocalized intensely with Aβ plaques in TgAD mice (row 1) and TgAD/HuPrP mice (row 2). TgAD/GSS mice exhibited no, or extremely low, PrP^A116V^ co-staining at the periphery of Aβ plaques (row 3). Large GSS-type plaques within the cerebellum typical of GSS plaques, were positive for PrP and not Aβ (row 4). TgAD/PrP^−/−^ mice show Aβ staining that colocalizes with the nuclear marker, DAPI (row 5). Scale bar = 10 μm. (**B**) Co-immunoprecipitation of Aβ and PrP from brain homogenates of ~4.2 month-old TgAD, TgAD/PrP^−/−^, and TgAD/GSS mice, and N2aAPPswe mouse neuroblastoma cell lysates from cells transfected with non-silencing siRNA (CTL), cells transfected with siRNA to knock down endogenous PrP^C^ (PrP^−^), and cells co-transfected with siRNA and a PrP^A116V^-containing pCB6 expression vector. To confirm binding from the input, 30 μg of protein from each sample was separated on SDS-PAGE and probed with human Fab D13 antibody to detect PrP (top panel). For co-IP, the samples were mixed with mouse anti-PrP SAF-32 antibody and the eluates were probed with D13 or rabbit anti-Aβ42 antibody PA3-16761. ɑ-tubulin was used as a loading control. The top panel blots of PrP from Tg mouse brain and N2a-APPswe cell lysates were each re-probed with α-tubulin to assess protein loads. The blots that label PrP after co-IP were re-probed for Aβ and displayed below their respective blot. (**C**) ELISA measurements of PrP concentrations in wild type (WT), TgPrP^−/−^, TgGSS, and TgHuPrP mouse brains (see Methods). *p < 0.05, **p < 0.01, ANOVA and post hoc multiple comparisons test. Each bar represents the mean ± S.D. (n = 6 mouse brains per group). (**D**) ELISA measurements (mean ± S.D) of brain-derived PrP binding to Aβ peptide. Aβ_42_ peptide (1.4 μM) was coated in a 96-well microplate (70 pmol per well) and the amount (pmol) of PrP bound was measured following application of 10% brain homogenates prepared from WT, TgPrP^−/−^, TgGSS, and TgAD/HuPrP mice, as described in Methods (n = 6 brains from each group at 3 dilutions per experiment, 2 independent experiments). *p < 0.05, **p < 0.01, ANOVA and multiple comparisons post-test. (**E**) Binding of PrP to Aβ, relative to the binding of mouse PrP^C^ from WT mice, corrected for the different expression levels of PrP in each mouse line. Samples as in D. **p < 0.01, ANOVA and post-test multiple comparisons, *p* < 0.01 between all groups, except *p* > 0.05 between TgPrP^−/−^ control and TgGSS.

PrP is known to colocalize with Aβ plaques in humans. We determined if PrP^C^ and PrP^A116V^ similarly colocalized with Aβ plaques, based on their comparable augmentation of Aβ plaque deposition in TgAD/GSS and TgAD/HuPrP mice. Confocal fluorescence microscopy was performed on brain sections dual-labeled for Aβ and PrP. We initially examined 100 consecutive Aβ plaques from each mouse line. In TgAD mice,  ~100% of Aβ plaques exhibited intense and homogeneous co-staining of PrP (Fig. 3A, row 1). This same staining pattern and 100% colocalization was observed in TgAD/HuPrP mice (Fig. 3A, row 2). In contrast, PrP^A116V^ primarily localized to the fringes of Aβ plaques and with a significantly lower signal intensity in TgAD/GSS mice (Fig. 3A, row 3). Although difficult to observe at low magnification, we were able to detect PrP^A116V^ labeling in as many as 75% of Aβ plaques. Within the cerebellum, large extracellular PrP^A116V^ - positive plaques displayed no Aβ immunoreactivity even at high magnification and gain (Fig. 3A, row 4). The absence of signal in TgAD/PrP^−/−^ mice confirmed the specificity of PrP antibody staining (Fig. 3A, row 5).

The above observations suggested a reduced or altered interaction between PrP^A116V^ and Aβ, compared with that between PrP^C^ and Aβ. This was further assessed with co-immunoprecipitation (co-IP) studies. Brain samples from TgAD and TgAD/GSS mice were assessed in parallel with mouse neuroblastoma (N2a) cells stably transfected with human APP carrying the Swedish mutation (N2aAPPswe)^29^ and modified to express PrP as in the Tg mouse lines. N2a-APPswe cells were transfected with either a non-silencing siRNA as control (N2A-APPswe-CTL) or active siRNA against *Prnp* to knock down endogenous PrP^C^ (N2a-APPswe-PrP^−^) or they were co-transfected with anti-PrP^C^ siRNA and a pCB6 expression vector containing mouse PrP^A116V^ (N2a-APPswe-PrP^A116V^), thereby modeling TgAD, TgAD/PrP^−/−^, and TgAD/GSS mice, respectively. Samples were initially probed with D13, a human F(ab) anti-mouse PrP antibody, to ensure adequate reactivity (Fig. 3B, top panel). PrP^C^ and PrP^A116V^ were immunoprecipitated using anti-PrP monoclonal antibody (mAb) SAF-32 and the eluate was probed with an anti-Aβ antibody. Aβ was readily detectable as monomers and dimers in the co-IP eluate from TgAD mice and N2a-APPswe-CTL cells that express PrP^C^, but not from TgAD/GSS mice or N2a-APPswe-PrP^A116V^ cells (Fig. 3B). TgAD/PrP^−/−^ mice and N2a-APPswe-PrP^−^ cells that lack PrP^C^ confirmed selectivity of the co-IP.

We next used ELISA to assess the ability of mouse brain-derived PrP^C^ and PrP^A116V^ to bind Aβ peptide. To first determine the concentration of PrP^C^ and PrP^A116V^ in the brain of each mouse line, sandwich ELISA was performed using a standard dilution curve with recombinant PrP^C^. The concentration of PrP^C^ in WT mice was 217.2 ± 38.8 pmol/g wet tissue. In agreement with prior estimates, PrP^A116V^ in TgGSS mice was ~4.5 times that of PrP^C^ in WT mice (977.6 ± 54.1 pmol/g) and HuPrP^C^ in TgHuPrP mice was ~5 times the level of PrP^C^ (1060.0 ± 86.9 pmol/g) in WT mice (Fig. 3C). Brain homogenates from each mouse line were then applied to plate wells coated with 70 pmol of human Aβ_42_ peptide to measure the binding (pmol) of PrP^C^ from WT mice, PrP^A116V^ from TgGSS mice, and HuPrP^C^ from TgHuPrP mice. PrP^C^ binding from WT mice measured 8.4 ± 1.9 pmol whereas binding from TgHuPrP mouse brain was 72.7 ± 8.5 pmol and PrP^A116V^ binding from TgGSS mice measured only 4.1 ± 1.0 pmol compared to 2.4 ± 0.8 pmol measured in control Tg PrP^−/−^ mice (Fig. 3D). When corrected for differences in PrP expression among the mouse lines and normalizing measurements to WT mice (100 ± 19.4%), mouse PrP^A116V^ binding to Aβ_42_ was calculated to be 10.8 ± 1.5% of mouse PrP^C^ binding whereas human PrP^C^ from TgHuPrP mice was 177.4 ± 9.5% of mouse PrP^C^ binding to Aβ (Fig. 3E). These results demonstrate a clear difference in Aβ binding to the different PrP molecules such that human PrP^C^ binds more efficiently to Aβ than mouse PrP^C^ and mouse PrP^A116V^ binding to Aβ is roughly 90% lower than mouse PrP^C^ binding. Thus, the colocalization immunofluorescence, co-IP, and ELISA binding studies all support impaired interaction of PrP^A116V^ with Aβ compared to PrP^C^.

### Aβ accumulates with intraneuronal markers in the absence of PrP^C^

**Figure 4:**
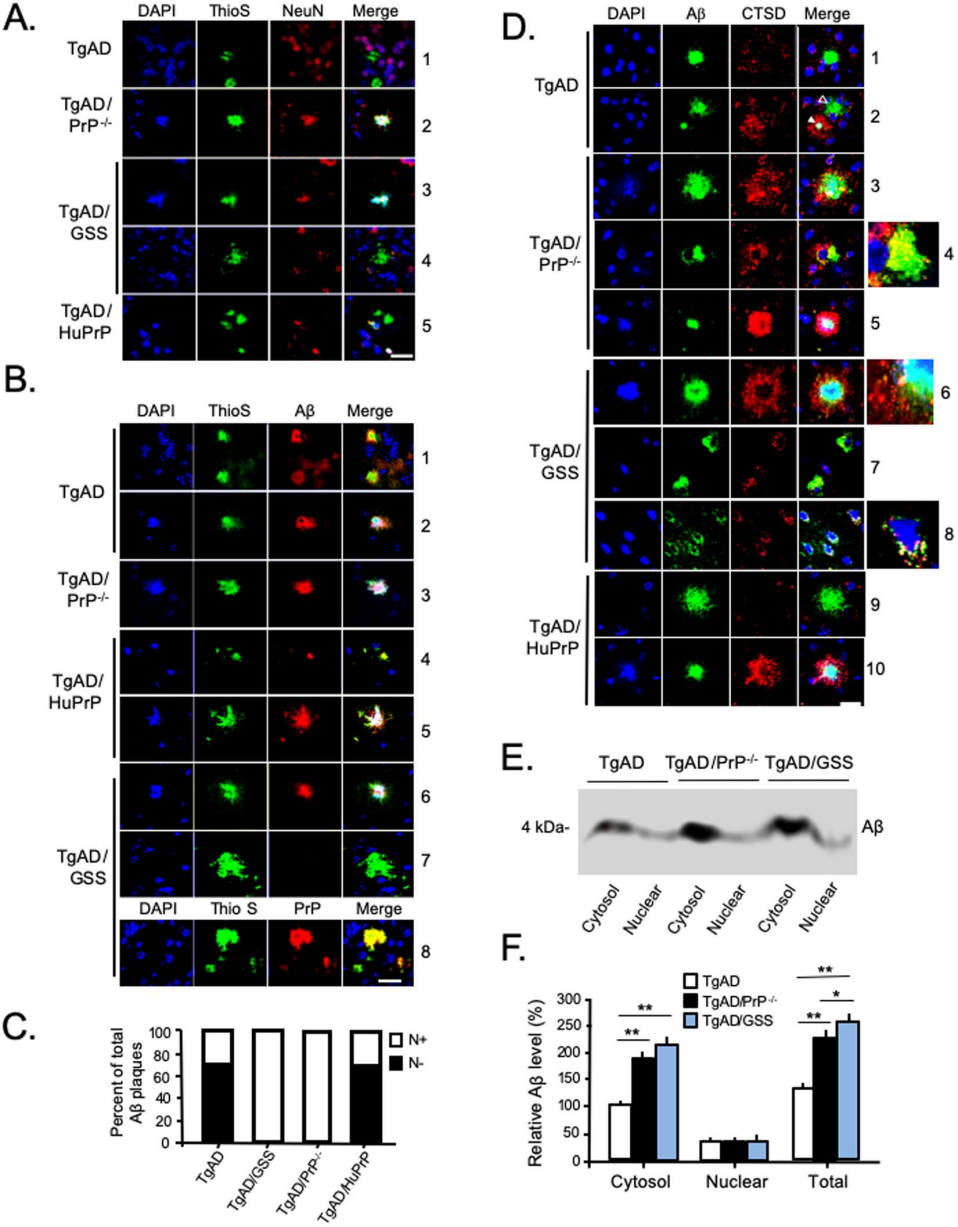
PrP^C^ expression correlates inversely with intracellular Aβ accumulation. (**A**) Confocal fluorescence (direct + indirect) images of paraffin embedded brain sections from the cortex of each Tg mouse line at ~4.2 months, stained with Thioflavin S (ThioS), DAPI, and anti-NeuN antibody. The most commonly associated plaque-types within each Tg mouse line are displayed. Spatially separated amyloid from NeuN or DAPI stained nuclei in TgAD mice (row 1). Amyloid deposits closely associated with NeuN and DAPI-positive nuclear material in TgAD/PrP^−/−^ mice (row 2). Nuclear marker associated amyloid in cerebrum (row 3) but not in cerebellum (row 4) of TgAD/GSS mice. Amyloid deposit distinct from NeuN or DAPI stained nuclei in TgAD/HuPrP mice (row 5). Original magnification × 100. Scale bar = 20 μm. (**B**) Mouse brain sections prepared as in (**A**), but stained with anti-Aβ antibody, ThioS, and DAPI. In TgAD mice Aβ plaques not associated with nuclear markers (row 1, lower plaque) were most prevalent whereas plaques associated with nuclear markers (row 2) were much less common. Nuclear marker-associated plaques were the only type seen in TgAD/PrP^−/−^ mice (row 3). Aβ plaques in TgAD/HuPrP mice werepredominantly separated from nuclear markers (row 4) although some  overlapped tightly with nuclear markers (row 5). Aβ deposits associated with nuclear markers in cerebrum of TgAD/GSS mice (row 6). Amyloid plaques in cerebellum of TgAD/GSS mice not associated with nuclear markers were not labeled by Aβ antibody (row 7) but were labeled by anti-PrP antibody (row 8). Original magnification × 100. Scale bar = 20 μm. (**C**) Bar graph displays the fraction of plaques not associated with nuclear markers (N−, solid bars) and those associated with nuclear markers (N+, open bars) in the four Tg mouse lines. The total area of N− and N+ Aβ plaques was determined using NIH ImageJ and plotted as the relative percentage measured for each Tg line at ~4.2 months of age. Each bar represents data from 3 parasagittal whole brain sections from each of 6 mice per group. See Supplemental Table 3A for actual values. (**D**) Representative brain sections prepared as in (**A**) but stained with anti-Aβ_42_ (MOAB-2) and anti-Cathepsin D (CTSD) antibodies, and DAPI. TgAD mice; row 1 - a well-formed extracellular Aβ plaque distinct from nuclear staining and not associated with CTSD staining, row 2 - a small Aβ plaque (solid arrowhead) within a CTSD-labeled cytosol and closely approximating nuclear material adjacent to a larger plaque not associated with nuclear staining or CTSD labeling (open arrowhead). TgAD/PrP^−/−^ mice; row 3 - a large Aβ plaque associated with nuclear staining surrounded by a dispersed CTSD-labeled cytosol, row 4 - a compact perinuclear Aβ plaque closely associated and partially colocalizing with CTSD-labeling, row 5 – a small plaque overlapping nuclear staining and surrounded by a CTSD densely-labeled cytosol. TgAD/GSS mice; row 6 - a dense plaque overlapping nuclear staining and surrounded by CTSD-labeled puncta, row 7- perinuclear accumulation of Aβ in cytosol with CTSD labeling in two cells, row 8- several cells with punctate Aβ within cytosol that colocalizes with CTSD puncta. TgAD/HuPrP mice; row 9 – Large extracellular Aβ-plaque distinct from nuclear material and CTSD-labeled puncta; row 10 – compact Aβ-plaque overlapping nuclear staining and surrounded by CTSD-labeled puncta. Enlarged sections from rows 4, 6, and 8 highlight colocalization of Aβ with CTSD-positive puncta. Original magnification 100X. Scale bar = 20 μm. (**E**) Representative Western blot of Aβ within cytosolic and nuclear fractions prepared from brain (see Methods) of TgAD, TgAD/PrP^−/−^ and TgAD/GSS mice. (**F**) Bar graph below the Western blot displays the mean relative level ± S.D. of cytosolic and nuclear Aβ normalized to cytosolic Aβ in TgAD mice (n = 3 brain samples/group). Semi-quantitation of signal density was performed using Quantity One software (Bio-Rad). Supplemental Table 3B lists the plotted values. ANOVA and post hoc multiple comparison tests were performed on each, *p < 0.05, **p < 0.01.

In patients with AD and in TgAD mice, Aβ plaques are typically extracellular. However, we noticed that plaques in TgAD/GSS mice were closely associated with neuronal nuclear markers. We questioned whether PrP^A116V^ expression modifies the location of Aβ deposits relative to the extra- and intra- cellular space. We assessed the localization of amyloid deposits relative to cell bodies in brain sections prepared from ~4.2 month-old TgAD, TgAD/PrP^−/−^, TgAD/GSS, and TgAD/HuPrP mice, using a triple-fluorescence staining protocol that included Thioflavin S (Thio S) to label amyloid, anti-NeuN antibody to label neuronal cell bodies, and DAPI to label nuclei. In TgAD mice, the majority of plaques were distinctly separated from cell bodies (Fig. 4A, row 1), suggesting these to be extracellular, while a smaller fraction was found in close proximity to nuclear markers. In contrast, most plaques in TgAD/PrP^−/−^ mice overlapped with NeuN and DAPI staining (Fig. 4A, row 2). That same pattern was evident in the majority of plaques in TgAD/GSS mice (Fig. 4A, row 3), with the exception of the large extracellular plaques within the cerebellum, presumed to be GSS plaques composed of PrP^A116V^ (Fig. 4A, row 4). TgAD/HuPrP mice exhibited both types of plaques in a similar proportion as in TgAD mice (Fig. 4A, row 5). We repeated the above analysis on adjacent brain sections using an anti-Aβ_42_ specific antibody (PA3-16761). This labeled nearly 100% of Thio S positive plaques in each mouse line, with the exception of the large cerebellar GSS plaques in TgAD/GSS mice (Fig. 4B). The distribution of Aβ staining paralleled Thio S staining such that the majority of Aβ staining in TgAD and TgAD/HuPrP mice was separated from DAPI staining of nuclei (Fig. 4B, rows 1 and 4) whereas a smaller fraction overlapped with it (Fig. 4B, rows 2 and 5). In contrast, essentially all plaques overlapped with DAPI staining in TgAD/PrP^−/−^ mice (Fig. 4B, row 3). The vast majority of plaques in TgAD/GSS mice also overlapped with DAPI staining (Fig. 4B, row 6) except for the large cerebellar plaques. The latter were detected primarily within the granule cell layer and were not labeled by Aβ antibody (Fig. 4B, row 7) but were immunostained by anti-PrP antibody (Fig. 4B, row 8), confirming these to be PrP plaques described in TgGSS mice^17^.

The ratio of Aβ plaques that did (N+) and did not (N−) overlap with DAPI nuclear staining in each mouse line was determined. Using an ImageJ plug-in, the mean area labeled by Aβ antibody relative to whole brain section for each plaque type was measured (Fig. 4C). At ~4.2 months the ratio of plaques in TgAD mice that did not overlap with DAPI compared to those that did (N−/N+) was 66.5/33.5. In TgAD/HuPrP mice the N−/N+ ratio was similar, at 69/31. Thus, roughly two-thirds of Aβ plaques in mice expressing either mouse or human PrP^C^, regardless of the level of expression, were extracellular. In contrast, virtually 100% of Aβ plaques were associated with nuclear markers in end-stage (~4.2 month-old) TgAD/GSS mice and in age-matched TgAD/PrP^−/−^ mice (Fig. 4C).

To support the intracellular location of Aβ, we co-labeled brain sections from each Tg mouse line at ~4.2 months of age with Aβ and Cathepsin D (CTSD), a marker for lysosomes (Fig. 4D). For this we used the MOAB-2 antibody, a highly Aβ-specific mAb that preferentially detects Aβ_42_ over Aβ_40_ and lacks cross-reactivity with APP or APP C-terminal fragments^30^. Two major patterns of plaque staining were observed; 1) Aβ distinct from both nuclear staining and CTSD-labeled puncta, supporting an extracellular location, were primarily observed in TgAD (Fig. 4D, row 1 and row 2-open arrowhead) and TgAD/HuPrP mice (row 9), and; 2) Aβ associated with nuclear staining and surrounded by, or colocalized with, CTSD-positive puncta, suggesting an intracellular pattern were predominantly observed in TgAD/PrP^−/−^ (rows 3–5) and TgAD/GSS (rows 6–8) mice, although a smaller number were seen in TgAD (row 2-solid arrowhead) and TgAD/HuPrP (row 10). Using these markers, roughly 60% of plaques in TgAD mice and 57% in TgAD/HuPrP mice were characterized as extracellular whereas ~100% in TgAD/PrP^−/−^ and TgAD/GSS mice were labeled as intracellular.

Although there appeared to be considerable overlap of Aβ staining with nuclear markers, in many cases a clear perinuclear or cytosolic accumulation was evident. We therefore separated nuclear and cytosolic fractions prepared from fresh brain samples of TgAD, TgAD/PrP^−/−^, and TgAD/GSS mice, to assess the relative distribution of Aβ within these two fractions (Fig. 4E). Aβ was predominantly found within the cytosolic fraction of each mouse line and although a similar level of Aβ was detected within the nuclear fraction among the different mouse lines, a significant increase in Aβ was measured within the cytosolic fraction in TgAD/GSS and TgAD/PrP^−/−^ mice relative to TgAD mice (Fig. 4F), supporting the intracellular accumulation of Aβ in the absence of PrP^C^.

**Figure 5:**
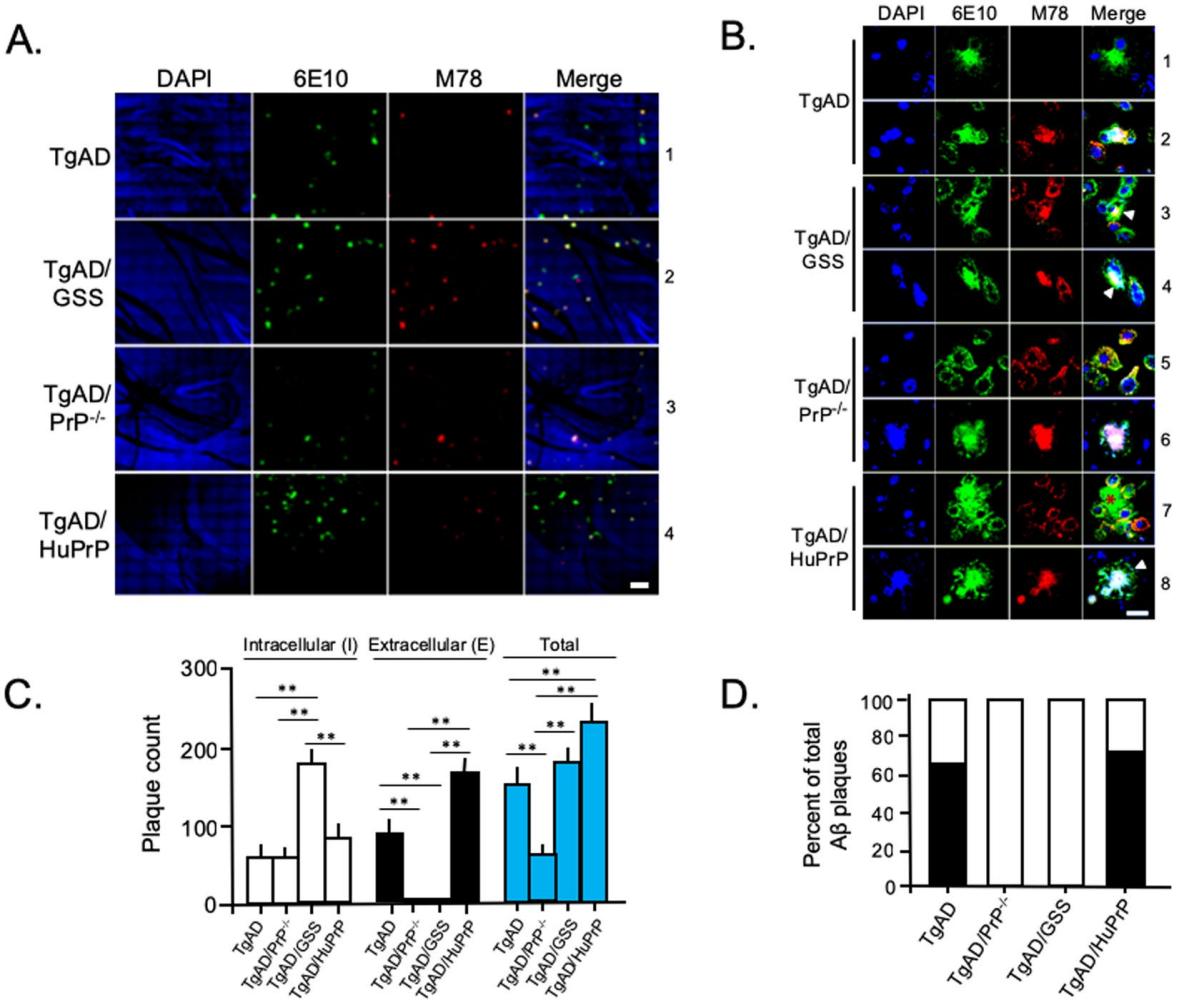
Intracellular-type Aβ plaque accumulation in mice lacking PrP^C^ is not reversed by PrP^A116V^. (**A**) Confocal fluorescence (direct + indirect) images of paraffin embedded mouse brain sections from the cortex of ~4.2 month-old Tg mouse lines. Sections were immunostained with M78 antibody to label intracellular fibrillar Aβ_42_, and 6E10 antibody to label intracellular and extracellular Aβ. Nuclei are labeled by DAPI. Separate channels and merged images are displayed. Yellow color denotes colocalization. Scale bar = 100 μm. (**B**) Representative confocal images (100X magnification) of brain sections from ~4.2 month-old Tg mice from panel A co-immunostained with 6E10 and M78. DAPI labeled nuclei. Extracellular Aβ plaques labeled only by 6E10 in TgAD mice (row 1) and TgAD/HuPrP mice (row 7, asterisk). Intracellular accumulation of Aβ dual-labeled with 6E10 and M78 as plaque-like accumulations in TgAD (row 2), TgAD/GSS (rows 3 and 4), mice, or diffuse accumulations, as in the example in TgAD/PrP^−/−^ (row 5) and TgAD/HuPrP (row 7). Arrowheads in merged images in rows 3, 4, and 8 indicate 6E10 labeling of the plasma membrane that circumscribes the co-stained intracellular accumulations. Scale bar = 10 μm. (**C**) Graphic display of actual mean ± S.D. of intracellular (open bars), extracellular (black bars), and total (blue bars) Aβ plaque counts per section for each of the four Tg mouse lines. Plaques labeled only by 6E10 were manually counted as extracellular and those stained by both M78 and 6E10 were counted as intracellular. Six mouse brains per line, 3 parasagittal sections per brain, were analyzed. See Supplemental Table 4A for actual values. (**D**) Graphic display depicts the fraction of intracellular (open bars) and extracellular (solid bars) Aβ plaques within each of the four Tg mouse lines. See Supplemental Table 4B for actual values.

We next employed the well-characterized M78 anti-fibrillar Aβ antibody that recognizes a discontinuous Aβ epitope specific to intraneuronal Aβ fibrils^25^. When used in combination with 6E10 antibody that labels Aβ regardless of its location, we detected plaques that were either labeled only by 6E10, suggesting an extracellular location, or co-labeled by 6E10 and M78, supporting an intraneuronal location (Fig. 5). We found the number of plaques labeled by 6E10 alone far outnumbered those co-labeled by 6E10 and M78 in TgAD mice (Fig. 5A, row 1). In contrast, the vast majority of plaques in TgAD/GSS mice were co-labeled by both antibodies (Fig. 5A, row 2). This same pattern was evident in ~4.2 month-old TgAD/PrP^−/−^ mice (Fig. 5A, row 3) whereas the majority of plaques in TgAD/HuPrP mice were labeled only by 6E10, paralleling the results in TgAD mice (Fig. 5A, row 4). High magnification (100X) images of single- and dual-labeled plaques from each mouse line confirmed the tight association between M78 and 6E10 signal, appearing plaque-like and often overlapping with DAPI-positive nuclear material (Fig. 5B, rows 2, 6 and 8) or as cytosolic accumulations, presumably as precursors to plaque formation (Fig. 5B, rows 3, 4, 5, and 7). In some cases, M78 labeling was associated with a combination of diffuse Aβ staining and an early stage perinuclear plaque **(**Fig. 5B. rows 3 and 4). In all cells in which Aβ was co-labeled with 6E10 and M78 antibodies, the plasma membrane could be visualized by 6E10 labeling of membrane-bound APP (Fig. 5B, rows 3, 4, 8 arrowheads), a feature clearly absent in extracellular plaques labeled only by 6E10 (Fig. 5B, rows 1 and 7-asterisk). Individual plaques were counted and plotted as the fraction co-labeled with M78 and 6E10 (intracellular) versus those labeled only by 6E10 (extracellular). The mean total Aβ plaque count per whole brain section correlated directly with the expression level of either PrP^C^ or PrP^A116V^ and the fraction of plaques co-labeled with M78 and 6E10 was significantly higher in mice lacking PrP^C^, whether or not they expressed PrP^A116V^ (Fig. 5C). When plotted as the fraction of intracellular vs extracellular plaques within each mouse line, roughly two-thirds of all Aβ plaques were 6E10 positive (i.e. extracellular) in TgAD and TgAD/HuPrP mice, whereas nearly 100% of Aβ plaques were 6E10 and M78 positive (i.e. intracellular) in TgAD/PrP^−/−^ and TgAD/GSS mice (Fig. 5D).

### PrP^C^ enhances secretion of Aβ in neuroblastoma cells expressing APPswe

**Figure 6:**
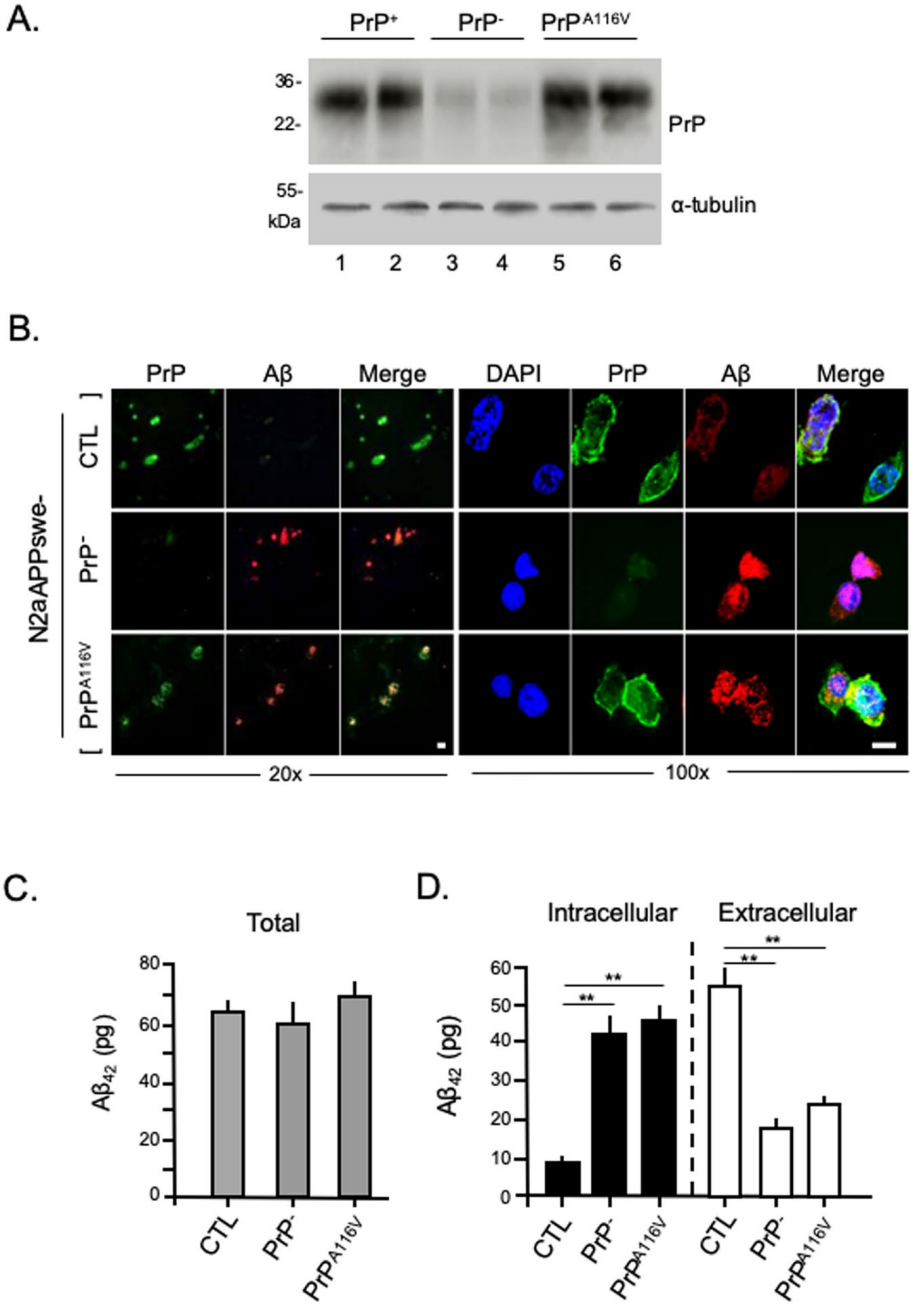
PrP^C^ but not PrP^A116V^ promotes secretion of Aβ from N2aAPPswe cells. (**A**) Western blot confirms PrP^C^ knockdown and PrP^A116V^ expression in N2aAPPswe cells used in Aβ secretion studies. Cells were transfected with a non-silencing control siRNA as control (CTL) (lanes 1 and 2), or they were transfected with anti-PrP^C^ siRNA (lanes 2 and 3), or co-transfected with anti-PrP^C^ siRNA and a pCB6 expression vector containing PrP^A116V^ (lanes 5 and 6). The transfection media was removed 24 h later and the cells were incubated with OPTI-MEM I for 24 h, then lysed and harvested for Western blotting. From cell lysates, 30 μg of protein was subjected to 12.5% SDS-PAGE then transferred to PVDF membranes and probed with anti-PrP SAF-32 mAb and α-tubulin. (**B**) N2aAPPswe cells were grown on coverslips and transfected as in A, then prepared for immunofluorescence and labeled with anti-PrP mAb SAF-32 and anti-Aβ_42_ antibody PA3-16761. Confocal fluorescence images of cells are shown at 20X (left panel) and 100X (right panel) magnification. Scale bars = 10 μm. (**C**) Bar graph displays ELISA measurements (pg) of total Aβ from control N2aAPPswe cells (CTL), after PrP knock down (PrP^−/−^), and after PrP knock down combined with PrP^A116V^ transfection. Samples used were 30 μg of protein from lysates and 5 μL of 10 mL media. Total human Aβ_42_ levels were calculated from the measures of total lysate and total media. Six samples per group with 2 replicates per sample were tested. See Supplemental Table 5 for actual values. ANOVA *p* > 0.05. (**D**) Intracellular (solid bars) and secreted (open bars) Aβ (pg) measured by ELISA from a fraction of cell lysate and media, respectively. Six samples per group with 2 replicates each were tested. See Supplemental Table 5 for actual values. ANOVA results for intracellular Aβ in cell lysates: *p* < 0.01, post hoc multiple comparisons test, **p < 0.01 between CTL and PrP^−^, CTL and PrP^A116V^; *p* > 0.05 between PrP^−^ and PrP^A116V^ cells. Aβ in media, *p* < 0.01 ANOVA, post hoc multiple comparisons test, **p < 0.01 between CTL and PrP^−^, and PrP^A116V^; *p* > 0.05 between PrP^−^ and PrP^A116V^ cells.

Based on the above results that suggest the loss of PrP^C^ rather than the expression of PrP^A116V^ leads to the accumulation of Aβ within an intraneuronal compartment, we questioned whether PrP^C^ promotes the secretion of Aβ. Immunofluorescence staining and ELISA were used to assess the relative proportions of intracellular and extracellular Aβ in N2aAPPswe cells modified to express Aβ and PrP to model the expression pattern of Tg mouse lines. Thus, N2a-APPswe cells were transfected with non-silencing control siRNA (N2aAPPswe-CTL) to model TgAD mice, or transfected with anti-PrP^C^ siRNA (N2aAPPswe-PrP^−^) to model TgAD/PrP^−/−^ mice, or co-transfected with anti-PrP^C^ siRNA and an expression vector containing PrP^A116V^ (N2aAPPswe-PrP^A116V^) to compare with TgAD/GSS mice. PrP^C^ knock-down and PrP^A116V^ expression were confirmed by Western blot (Fig. 6A). Cell staining revealed intracellular accumulation of Aβ in N2aAPPswe-PrP^−^ and N2aAPPswe-PrP^A116V^ cells, compared with N2aAPPswe-CTL cells (Fig. 6B). ELISA was used to measure Aβ_42_ in cell lysates and media (Fig. 6C,D). Total Aβ was similar among the different cell treatments, although we noted a slight trend for a reduction in total Aβ in N2a-APPswe-PrP^−^ cells and an increase in N2a-APPswe-PrP^A116V^ cells, compared with N2a-APPswe-CTL cells (Fig. 6C). However, the distribution of Aβ shifted from predominantly within the media of N2a-APPswe-CTL cells to predominantly intracellular in N2a-APPswe-PrP^−^ cells (Fig. 6D). A similar increase in intracellular Aβ and a reduction in extracellular Aβ was observed in N2aAPPswe-PrP^A116V^ cells, supporting the facilitation of Aβ secretion by PrP^C^ but not by PrP^A116V^ (Fig. 6D).

### PrP^C^ but not PrP^A116V^ is co-secreted with Aβ in exosomes

**Figure 7:**
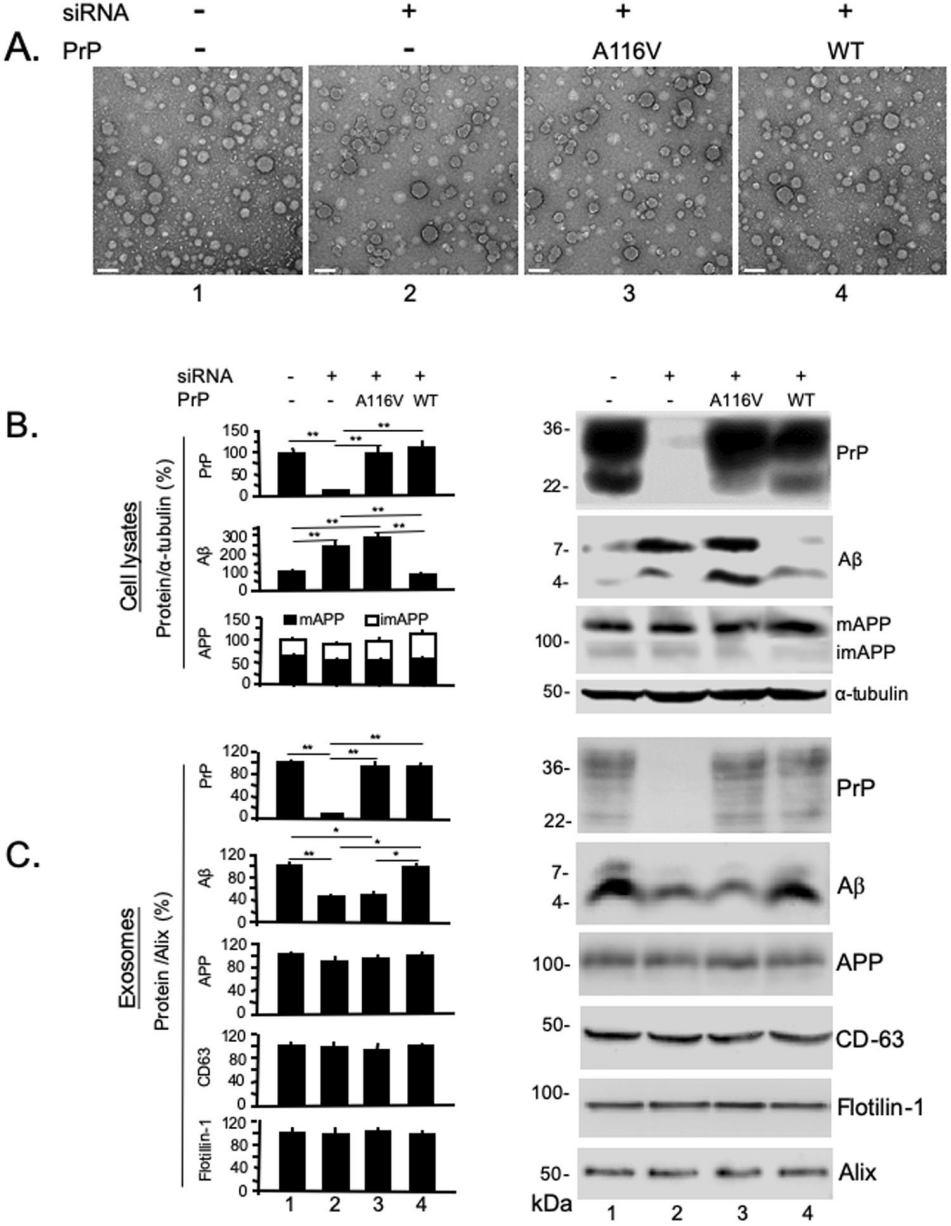
PrP^C^ but not PrP^A116V^ is co-secreted with Aβ in exosomes. (**A**) Transmission electron microscopy (TEM) of exosomes isolated from media of N2a-APPswe cells mock-transfected (1), transfected with siRNA against PrP^C^ (2), siRNA against PrP^C^ plus pCB6-PrP^A116V^ (3), or siRNA against PrP^C^ plus pCB6-WT-PrP (4). Exosomes were isolated and purified by ExoQuick-TC ULTRA EV Isolation Kit for Tissue Culture Media (SBI), then applied to copper grids, stained with uranyl acetate prior to visualization with TEM. Vesicular morphology in the size range of ~20–70 nm is consistent with exosomes and each prep had similar densities. (**B**,**C**) Semi-quantitation of PrP, Aβ, and APP in cell lysates and exosomes prepared from N2a-APPswe cells. Representative Western blots are adjacent to the corresponding bar graph. Cells were harvested 48 h after transfection and exosomes prepared as described in Methods. Lane 1) mock-transfected, Lane 2) transfected with anti-PrP^C^ siRNA, Lane 3) cotransfected with anti-PrP^C^ siRNA and pCB6 vector carrying PrP^A116V^, Lane 4) cotransfected with anti-PrP^C^ siRNA and pCB6 vector carrying WT PrP^C^. Antibodies were SAF-32 (PrP), anti-Alix mAb, anti-flotillin-1 mAb, anti-CD63 mAb, anti-Aβ42 antibody, 22C11 (APP), and α-tubulin antibody. To detect Aβ, preps were loaded onto 6% and 16.5% discontinuous tricine-tris SDS-PAGE gels with SDS loading buffer without β-ME, otherwise 12% SDS gels with β-ME. PrP, APP and α-tubulin were individually probed on the same blot and displayed separately for cell lysates and exosome fractions. Because a discontinuous gel was necessary to probe for Aβ, it was run on a separate Western and re-probed for α-tubulin from cell lysates. Westerns of exosome samples were initially probed for Aβ, then reprobed for flotillin-1, alix, and CD63. Signal Intensities of PrP, APP, Aβ, CD63, and flotillin-1 were normalized to signals of α-tubulin (cell lysates) or Alix (exosomes) and presented as the percentage change from untreated N2a-APPswe cells. Bars are means ± S.D. of 3 independent experiments. See Supplemental Tables 6A (lysates) and 6B (exosomes) for actual values plotted. ANOVA and post hoc multiple comparisons test, *p < 0.05 and **p < 0.01.

Exosomes are membrane bound organelles derived from multivesicular bodies (MVB) that deliver intracellular debris and cytosolic protein aggregates to the extracellular space^31^. Both Aβ and PrP have been shown to be secreted via exosomes prepared from N2a and N2a-APPswe cells^32–34^. We questioned whether the tight relationship we found between Aβ and PrP^C^ persisted until their release in exosomes. To assess this, we used the ExoQuick-TC ULTRA EV Isolation Kit to isolate and purify exosomes from the media of N2aAPPswe-CTL cells and from those following PrP^C^ knock down or PrP^C^ knock down followed by transient expression of either PrP^A116V^ or WT PrP^C^. Each prep was visualized by transmission electron microscopy (TEM), which revealed numerous vesicles ranging in size from ~20 to 70 nm in each treatment group (Fig. 7A). The presence and relative levels of PrP^C^, PrP^A116V^, Aβ, and APP in exosomes was next assessed by Western blotting and semi-quantification by densitometry (Fig. 7B,C). Antibodies against Alix, flotillin-1, and the more specific tetraspainin CD-63, were used to confirm exosome-containing cellular fractions^32,33,35,36^. Cell lysates were prepared in parallel and tested for PrP and Aβ. Expression levels of recombinant PrP^A116V^ and WT PrP were similar to endogenous PrP^C^ levels in cell lysates (Fig. 7B) and exosomes (Fig. 7C), suggesting similar proportions of each were secreted via exosomes (Fig. 7C). PrP^C^ levels were knocked down more than 80% of endogenous levels after siRNA (Fig. 7B, lane 2). In lysates from PrP^C^ knocked-down cells and knocked-down cells transiently expressing PrP^A116V^, Aβ monomers and dimers were significantly increased (Fig. 7B, lanes 2 and 3), relative to control N2aAPPswe cells expressing endogenous PrP^C^ or in PrP^C^ knocked-down cells transiently expressing recombinant WT PrP^C^ (Fig. 7B, lanes 1 and 4). Exosomes prepared from PrP^C^ knocked-down cells had significantly reduced Aβ dimers and monomers, compared to control cells (Fig. 7C, lane 2), consistent with the intracellular retention of Aβ. When PrP^A116V^ was expressed in cells with endogenous PrP^C^ knocked down, a similarly low level of Aβ was recovered from exosomes (Fig. 7C, lane 3), whereas recombinant expression of WT PrP^C^ in the absence of endogenous PrP^C^ restored Aβ to control levels (Fig. 7C, lane 4). Although we did detect APP in exosomes, the levels were relatively low and did not vary among the different cell treatments, suggesting a direct effect of PrP on Aβ secretion rather than an indirect effect on APP (Fig. 7C).

## Discussion

TgAD/GSS mice were generated to better assess whether *in vivo* amyloid cross-seeding occurs between PrP and Aβ. We reasoned that the highly amyloidogenic PrP^A116V^ would more effectively promote Aβ plaque deposition than PrP^C^ in a mouse model of AD. The increased burden of both Aβ and PrP plaques relative to age-matched single-disease TgAD and TgGSS mice strongly supports cross-talk between these two diseases. However, our results question cross-seeding as the principal mechanism. Although PrP^C^ intensely colocalized with Aβ plaques and it co-immunoprecipitated with Aβ from mouse brain and N2aAPPswe cells, ostensibly supporting cross-seeding, the highly amyloidogenic PrP^A116V^ only weakly labeled the fringes of Aβ plaques and did not co-immunoprecipate with Aβ, at least at an easily detectable level. Furthermore, Aβ was not detected in GSS plaques by immunofluorescence staining and the binding of PrP^A116V^ to Aβ was 90% less than PrP^C^ in an ELISA-based binding assay. Despite the obvious disparity in their interaction with Aβ, PrP^C^ and PrP^A116V^ increased Aβ plaque burden equally, suggesting that the more amyloidogenic PrP^A116V^ provides no additional Aβ promoting effect over that of PrP^C^. In fact, a direct correlation between PrP expression levels and the level of Aβ and Aβ plaque burden was clearly demonstrated by our Tg mouse lines expressing varied levels of PrP.

Although cross-seeding cannot be ruled out by these studies, our results suggest an indirect mechanism might contribute to the promotion of AD by PrP. Indirect actions of PrP to affect AD are not without precedent. Parkin *et al*.^5^ initially reported a suppressive effect of PrP^C^ on BACE1, the enzyme responsible for the first cleavage of APP that initiates the amyloidogenic cascade to generate Aβ. PrP was proposed to bind to and sequester BACE1 from reaching the plasma membrane where APP is cleaved. However, BACE1 suppression by PrP would contrast with our findings, as higher levels of PrP would inhibit Aβ production. Interestingly, a subsequent report found that PrP expression promoted BACE1 expression in SH-SY5Y cells^37^, which better aligns with our observations. Although we did not assess BACE1 expression, we did find that steady state levels of APP correlated with PrP expression levels, thereby providing another possible source for the increase in Aβ plaques. Ordonez-Gutierrez *et al*.^38^ did not report an increase in APP in AD mice over-expressing PrP^C^ but Kralovicova *et al*.^39^ reported a two-fold increase in APP in tga20 mice that over-express PrP^C^ ~8X and a reduction of APP in PrP^C^ knock-out mice. We did not address the mechanism(s) by which PrP might affect steady state levels of APP. Based on the varied effects of PrP and its multiple potential binding partners^40,41^ there may be a variety of pathways by which it indirectly affects APP levels and increases Aβ. For example, the ability of PrP to bind and deliver copper to cells^42^ might contribute to an indirect increase in APP levels, based on evidence that APP expression depends, at least in part, on copper^43^. Thus, an increase in copper delivery by PrP might enhance expression of APP. PrP has also been shown to suppress autophagy^44^, a process known to promote clearance of Aβ^45^. Regardless of the specific mechanism, our findings provide new support to consider an indirect effect rather than cross-seeding by which PrP expression promotes Aβ plaque burden.

In addition to the above findings, our studies also revealed a new fundamental role of PrP^C^ as a facilitator of Aβ secretion via exosomes. We initially noticed that Aβ plaques were in close proximity to neuronal nuclei in TgAD/GSS mice in contrast to those in TgAD mice that were primarily distinct from cell bodies, as is expected for extracellular plaques. The vast majority of Aβ plaques in TgAD/GSS mice either colocalized or were closely associated with intraneuronal markers NeuN, DAPI, and CTSD, and nearly all were labeled by M78, the intraneuronal-selective Aβ antibody, whereas roughly two-thirds of Aβ plaques in PrP^C^ - expressing TgAD and TgAD/HuPrP mice were extracellular. Because TgAD/PrP^−/−^ and TgAD/GSS mice displayed the same pattern of plaque distribution, we conclude that the absence of PrP^C^ rather than the presence of PrP^A116V^, is responsible for the intraneuronal accumulation of Aβ. This conclusion is strengthened by the finding that PrP^A116V^ expressed in N2aAPPswe cells did not reverse the intracellular accumulation of Aβ or the impairment in delivery of Aβ to exosomes following PrP^C^ knock-down. This process was not linked to APP, as we found consistently low levels of APP in exosomes that did not vary with PrP^C^ expression. Our findings invite the hypothesis that PrP^C^ acts as a carrier protein that delivers toxic Aβ to the extracellular space, for release via exosomes. As a GPI-anchored membrane protein, this function of PrP^C^ appears tenable.

The impaired ability of PrP^A116V^ to deliver Aβ to exosomes is unlikely to result from a lack of contact with Aβ within cellular compartments, since both PrP^C^ and PrP^A116V^ follow the secretory pathway. While a small fraction of PrP^A116V^ acquires a transmembrane topology^46^, this should not completely eliminate a potential interaction. It is more likely that the A116V mutation disrupts a binding epitope for Aβ or it induces a change in PrP conformation that conceals it. In fact, a binding site for Aβ has been mapped to three overlapping segments of mouse PrP located between residues 95 and 118^13,47^. We found a significant reduction in binding of brain-derived PrP^A116V^ to Aβ, compared with brain-derived PrP^C^, further supporting a disruption of this binding epitope as the cause for the intracellular accumulation and lack of exosome delivery in TgAD/GSS mice. PrP^C^ has been shown to bind to Aβ monomers, fibrils and Aβ-derived diffusible ligands (ADDLs) with different affinities^48^, but in our hands Aβ monomers and dimers were easily detected following pull-down of PrP^C^.

Intracellular Aβ accumulation and fibril formation appear to be early events in AD pathogenesis^25,49^ and a source of neuritic amyloid plaque formation^25,50^, in addition to contributing to neuronal loss and impaired glutamatergic synaptic function^51^. It would seem intuitive that Aβ elimination via exosomes is beneficial to the neuron and the loss of this function would contribute to disease but evidence for this is currently lacking. Nonetheless, it is interesting to speculate that the increased cell death and acceleration of GSS in TgAD/GSS mice might possibly result from the intracellular accumulation of Aβ. This will be important to confirm, as it may provide greater insight into the role of intracellular Aβ in the pathophysiology of AD.

In summary, our findings provide additional layers of complexity regarding the role(s) of PrP in AD. We show that PrP^C^ and PrP^A116V^ are equally effective at increasing steady state APP and promoting Aβ plaque deposition, yet they have quite different profiles with respect to their interactions with Aβ. This latter feature could be the reason for their difference in ability to deliver Aβ to exosomes. We propose that the colocalization of PrP^C^ within Aβ plaques might be a byproduct of its carrier function and intraneuronal binding to a developing Aβ plaque. When Aβ is released into the extracellular space, PrP is already incorporated within the plaque. It is interesting to note that a previously reported role of PrP^C^ in AD is to function as a surface receptor for Aβ^6^ that leads to neuronal toxicity, whereas the carrier function we propose acts to eliminate intraneuronal Aβ and play a protective role^52^. However, these two roles are not mutually exclusive and they depend on a common property of PrP^C^ to bind Aβ, albeit at different steps in its cellular trafficking. Could PrP^C^ provide a balance between neurotoxic and neuroprotective pathophysiology in AD? If so, a better understanding of this balance might reveal additional targets for treatment of AD.

## Supplementary information

Supplemental Tables

Supplementary Information
