# Supplementary material for: “Dual Disease” TgAD/GSS mice exhibit enhanced Alzheimer’s disease pathology and reveal PrPC-dependent secretion of Aβ
Source: Sci Rep. 2019 Jun 12;9:8524. doi: 10.1038/s41598-019-44317-w (PMC6562043; doi:10.1038/s41598-019-44317-w)
Supplement: Supplementary file 1 — Supplemental Tables [file 41598_2019_44317_MOESM1_ESM.docx]

**Manuscript # SREP-18-28306-T**

**“Dual Disease” TgAD/GSS mice exhibit enhanced Alzheimer’s disease pathology and reveal PrP^C^-dependent secretion of Aβ**

Kefeng Qin, Lili Zhao, Crystal Gregory, Ani Solanki, and James A. Mastrianni*

**Supplemental Tables**

**Table 1. Disease stages and Aβ plaque burden in TgAD mouse lines**

**Table 1A.** Mean age (days) at each disease stage (from Fig. 1A)

| Stage | TgGSS (n=20) | TgAD/GSS (n=15) | *p* |
| --- | --- | --- | --- |
| A1 | 120.0±10.7 | 90.7±9.5 | 0.0001 |
| A2 | 132.1±12.7 | 106.1±6.0 | 0.0001 |
| A3 | 154.0±16.8 | 118.4±4.0 | 0.0001 |
| A4 | 164.9±16.7 | 122.0±5.1 | 0.0001 |
| A5/Death | 174.6±18.4 | 126.0±8.2 | 0.0001 |

Students *t-*test was used to determine *p* value.

**Table 1B.** Relative (%) Aβ plaque burden in ~4.2 month-old Tg mouse lines (from Fig. 1I)

| Mouse line | Relative (%) Aβ plaque burden |
| --- | --- |
| (1) TgAD | 100.0±9.2 |
| (2) TgAD/PrP^-/-^ | 42.3±5.7 |
| (3) TgAD/GSS | 130.1±11.2 |
| (4) TgAD/HuPrP | 136.0±12.5 |
| *p* | 0.000 |
| 1:2 | ** |
| 1:3 | ** |
| 1:4 | ** |
| 2:3 | ** |
| 2:4 | ** |
| 3:4 | - |

ANOVA post hoc MC test *p* values between groups indicated

**Table 2. AD and PrD related proteins in TgAD mouse lines**

**Table 2A.** Steady state levels of PrP or Aβ (relative to α-tubulin) in TgAD mouse lines (Fig. 2B, 2C, 2G and 2H)

| Mouse line | Protein/α-tubulin (%) | | | | | | |
| --- | --- | --- | --- | --- | --- | --- | --- |
|  | PrP | Aβ (PA3-16761) | PrP | APP | mAPP | imAPP | PS1 |
| (1) TgAD | 100.0±9.8 | 100.0±4.1 | 100.0±10.3 | 100.0±3.4 | 44.5±8.0 | 55.5±8.0 | 100.0±9.3 |
| (2) TgAD/PrP-/- | 0.0±0.0 | 39.2±14.1 | 0.0±0.0 | 48.0±1.9 | 48.0±1.9 | 22.7±11.1 | 25.3±9.3 |
| (3) TgAD/GSS | 359.4±37.2 | 133.3±9.3 | 320.2±33.8 | 177.1±27.2 | 80.9±27.3 | 96.2±27.8 | 105.5±11.1 |
| (4) TgAD/HuPrP | 353.3±34.8 | 145.1±315.7 | 337.5±27.9 | 160.2±23.2 | 75.2±25.7 | 84.9±20.5 | 111.4±16.5 |
| *p* | 0.000 | 0.000 | 0.000 | 0.000 | 0.023 | 0.000 | 0.321 |
| 1:2 | ** | ** | ** | - | - | ** | - |
| 1:3 | ** | ** | ** | ** | * | ** | - |
| 1:4 | ** | ** | ** | * | - | - | - |
| 2:3 | ** | ** | ** | ** | - | ** | - |
| 2:4 | ** | ** | ** | ** | - | ** | - |
| 3:4 | - | - | - | - | - | - | - |
| (5) TgGSS | 314.3±30.7 | 0.0±0.0 |  |  |  |  |  |
| (6) TgHuPrP | 318.6±36.5 | 0.0±0.0 |  |  |  |  |  |
| (7) TgPrP-/- | 0.0±0.0 | 0.0±0.0 |  |  |  |  |  |
| 3:5 | - | N/A |  |  |  |  |  |
| 4:6 | - | N/A |  |  |  |  |  |
|  | Fig. 2B | Fig. 2C | Fig. 2G | | | | Fig. 2H |

ANOVA was used to determine group *p* value followed by post hoc multiple comparisons (MC) test. * = *p*<0.05, ** = *p*<0.01, - = *p*>0.05. (analysis from 3 replicate Western blots from each of 3 mice per group).

**Table 2B.** Concentrations of FA-Aβ and RIPA-Aβ in TgAD mouse lines (Fig. 2D & E)

| Mouse line | FA-Aβ (pmol/g) | RIPA-Aβ (pmol/g) |
| --- | --- | --- |
| (1) TgAD | 765.3 ± 140.5 | 7.1 ± 2.0 |
| (2) TgAD/PrP^-/-^ | 493.4 ± 66.4 | 4.7 ± 0.7 |
| (3)TgAD/GSS | 1931.1 ± 288.4 | 20.6 ± 4.7 |
| (4) TgAD/HuPrP | 2,121.0 ± 465.1 | 22.1 ± 3.0 |
| *p* | 0.000 | 0.000 |
| 1:2 | * | * |
| 1:3 | ** | ** |
| !:4 | ** | ** |
| 2:3 | ** | ** |
| 2:4 | ** | ** |
| 3:4 | - | - |

ANOVA was used to determine *p* value in the group and then performed Post hoc multiple comparisons (MC) test. * = *p*<0.05, ** = *p*<0.01, - = *p*>0.05. (n = 3 mice per group, 3 replicates each). n = 3 brains per group. 3 replicate ELISA of for 1 brain sample from each of 3 mice per group.

**Table 3. Intracellular and extracellular Aβ plaques in TgAD mouse lines**

**Table 3A.** Relative Intracellular (N+) to Extracellular (N-) distribution of Aβ plaques in TgAD mice with differing PrPs (Fig. 4C)

| Mouse line | Percentage (%) of Aβ plaques | |
| --- | --- | --- |
|  | Intracellular (N+) | Extracellular (N-) |
| TgAD | 33.5±6.1 | 66.5±6.1 |
| TgAD/PrP^-/-^ | 100.0±0.0 | 0.0±0.0 |
| TgAD/GSS | 100.0±0.0 | 0.0±0.0 |
| TgAD/HuPrP | 30.9±7.3 | 69.1±7.3 |

n = 6 mice per group, 3 parasagittal whole brain sections per mouse analyzed

**Table 3B.** Cytosol/nuclear fraction of Aβ in TgAD mice with differing PrPs (Fig. 4F)

| Mouse line | Percentages (%) of Aβ | | |
| --- | --- | --- | --- |
|  | Cytosol | Nuclear | Total |
| (1) TgAD | 100.0 ± 13.4 | 32.3 ± 7.4 | 132.3 ± 7.4 |
| (2) TgAD/PrP^-/-^ | 189.9 ± 12.1 | 37.6 ± 0.9 | 227.5 ± 13.0 |
| (3) TgAD/GSS | 217.7 ± 15.9 | 36.8 ± 13.9 | 254.5 ± 29.3 |
| *p* | 0.000 | 0.755 | 0.001 |
| 1:2 | ** | - | ** |
| 1:3 | ** | - | ** |
| 2:3 | - | - | - |

Data is normalized to the cytosolic fraction of Aβ in TgAD mice (n = 3 mouse brains/group)

**Table 4. Intracellular and extracellular Aβ plaque counts in TgAD mice with differing PrPs**

**Table 4A.** Aβ plaque counts in Tg mouse lines incorporating AD-related transgenes (Fig. 5C)

| Mouse line | Aβ plaque counts | | |
| --- | --- | --- | --- |
|  | Intracellular | Extracellular | Total |
| (1) TgAD | 61.2±16.8 | 94.2±9.1 | 155.4±20.9 |
| (2) TgAD/PrP^-/-^ | 60.2±9.0 | 0.2±2.5 | 60.4±8.9 |
| (3) TgAD/GSS | 180.4±22.7 | 1.7±4.3 | 182.1±18.5 |
| (4) TgAD/HuPrP | 72.5±9.3 | 162.1±17.3 | 234.6±35.2 |
| *p* | 0.000 | 0.000 | 0.000 |
| 1:2 | - | ** | ** |
| 1:3 | ** | ** | - |
| 1:4 | - | ** | ** |
| 2:3 | ** | - | ** |
| 2:4 | - | ** | ** |
| 3:4 | ** | ** | ** |

n = 6 mice per group, 3 brain sections each

**Table 4B.** Percent Intracellular and Extracellular Aβ plaques in TgAD mice with differing PrPs (Fig. 5D)

| Mouse line | % of total Aβ | |
| --- | --- | --- |
|  | Intracellular | Extracellular |
| (1) TgAD | 39.4±5.1 | 60.6±7.0 |
| (2) TgAD/PrP-/- | 99.7±4.0 | 0.3±4.0 |
| (3) TgAD/GSS | 98.8±2.4 | 1.2±2.4 |
| (4) TgAD/HuPrP | 30.9±7.3 | 69.1±7.3 |
| *p* | 0.000 | 0.000 |
| 1:2 | ** | ** |
| 1:3 | ** | ** |
| 1:4 | - | - |
| 2:3 | - | - |
| 2:4 | ** | ** |
| 3:4 | ** | ** |

Calculated from data in Table 4A (n = 6 mice per group, 3 parasagittal whole brain sections per mouse)

**Table 5. ELISA measured Aβ concentrations in N2aAPPswe cells (Fig. 6C & D)**

| Aβ (μg) |  | CTL | PrP^-^ | PrP^A116V^ |  | ANOVA |  | Between group comparisons (MC test) | | |
| --- | --- | --- | --- | --- | --- | --- | --- | --- | --- | --- |
|  |  | (1) | (2) | (3) |  | *p* |  | 1:2 | 1:3 | 2:3 |
| Total |  | 64.5±10.5 | 60.5±14.2 | 70.5±11.9 |  | 0.389 |  | - | - | - |
| Intracellular |  | 9.4±2.4 | 42.2±10.4 | 46.1±7.8 |  | 0.000 |  | ** | ** | - |
| Extracellular |  | 55.1±9.2 | 18.4±4.7 | 24.4±4.3 |  | 0.000 |  | ** | ** | - |

MC = multiple comparisons; * = *p*<0.05, ** = *p*<0.01, - = *p*>0.05; n=6/group.

**Table 6. Relative levels of PrP, Aβ, and APP in N2a-APPswe cell lysates and exosomes.**

**Table 6A.** Semi-quantitation of PrP, Aβ, and APP in cell lysates (Fig. 7B)

| Protein |  | Protein/α-tubulin (%) | | | | ANOVA | post hoc MC test | | | | | |
| --- | --- | --- | --- | --- | --- | --- | --- | --- | --- | --- | --- | --- |
|  |  | N2aAPPswe-CTL | N2aAPPswe-PrP^-/-^ | N2aAPPswe-PrP^A116V^ | N2aAPPswe-PrP | *p* | 1:2 | 1:3 | 1:4 | 2:3 | 2:4 | 3:4 |
|  |  | (1) | (2) | (3) | (4) |  |  |  |  |  |  |  |
| PrP |  | 100.0±5.1 | 11.0±2.1 | 99.4±5.5 | 114.4±15.8 | 0.000 | ** | - | - | ** | ** | - |
| Aβ |  | 100.0±9.0 | 241.7±50.4 | 286.3±46.2 | 79.6±19.3 | 0.000 | ** | ** | - | ** | ** | - |
| APP |  | 100.0±8.5 | 91.3±6.6 | 96.9±14.7 | 115.3±7.2 | 0.081 | - | - | - | - | - | - |
| mAPP |  | 100.0±15.8 | 77.7±13.9 | 77.1±14.6 | 77.6±10.8 | 0.447 | - | - | - | - | - | - |
| imAPP |  | 100.0±29.8 | 105.5±43.1 | 122.5±62.6 | 171.4±18.5 | 0.223 | - | - | - | - | - | - |

n = 3 experiments in triplicate

**Table 6B**. Semi-quantitation of PrP, Aβ, APP and exosome markers in exosomes prepared from N2aAPPswe cells (Fig. 7C)

| Protein | Protein/Alix (%) | | | |  | ANOVA |  | Post hoc MC test | | | | | |
| --- | --- | --- | --- | --- | --- | --- | --- | --- | --- | --- | --- | --- | --- |
|  | N2aAPPswe-  CTL  (1) | N2aAPPswe-PrP^-/-^  (2) | N2aAPPswe-PrP^A116V^  (3) | N2aAPPswe-PrP  (4) |  | *p* |  | 1:2 | 1:3 | 1:4 | 2:3 | 2:4 | 3:4 |
| PrP | 100.0±4.0 | 0.0±0.0 | 103.8±20.8 | 93.0±12.2 |  | 0.000 |  | ** | - | - | ** | ** | - |
| Aβ | 100.0±15.9 | 35.8±4.9 | 38.8±7.2 | 97.2±22.6 |  | 0.001 |  | ** | ** | - | - | ** | ** |
| APP | 100.0±5.8 | 97.3±18.1 | 105.6±5.0 | 97.8±7.6 |  | 0.808 |  | - | - | - | - | - | - |
| CD63 | 100.0±14.2 | 100.4±2.3 | 106.5±4.3 | 100.2±4.4 |  | 0.705 |  | - | - | - | - | - | - |
| Flotillin-1 | 100.0±4.9 | 104.6±5.8 | 106.9±5.0 | 103.3±4.8 |  | 0.464 |  | - | - | - | - | - | - |

MC = multiple comparisons; * = *p*<0.05, ** = *p*<0.01, - = *p*>0.05; (n = 3 experiments in triplicate)
