# Supplementary material for: “Dual Disease” TgAD/GSS mice exhibit enhanced Alzheimer’s disease pathology and reveal PrPC-dependent secretion of Aβ
Source: Sci Rep. 2019 Jun 12;9:8524. doi: 10.1038/s41598-019-44317-w (PMC6562043; doi:10.1038/s41598-019-44317-w)
Supplement: Supplementary file 2 — Supplementary Information [file 41598_2019_44317_MOESM2_ESM.pdf]

Supplementary Information:

"Dual Disease" TgAD/GSS mice exhibit enhanced Alzheimer's disease pathology and reveal PrP^{C}-dependent secretion of Aβ

Kefeng Qin, Lili Zhao, Crystal Gregory, Ani Solanki, and James A. Mastrianni

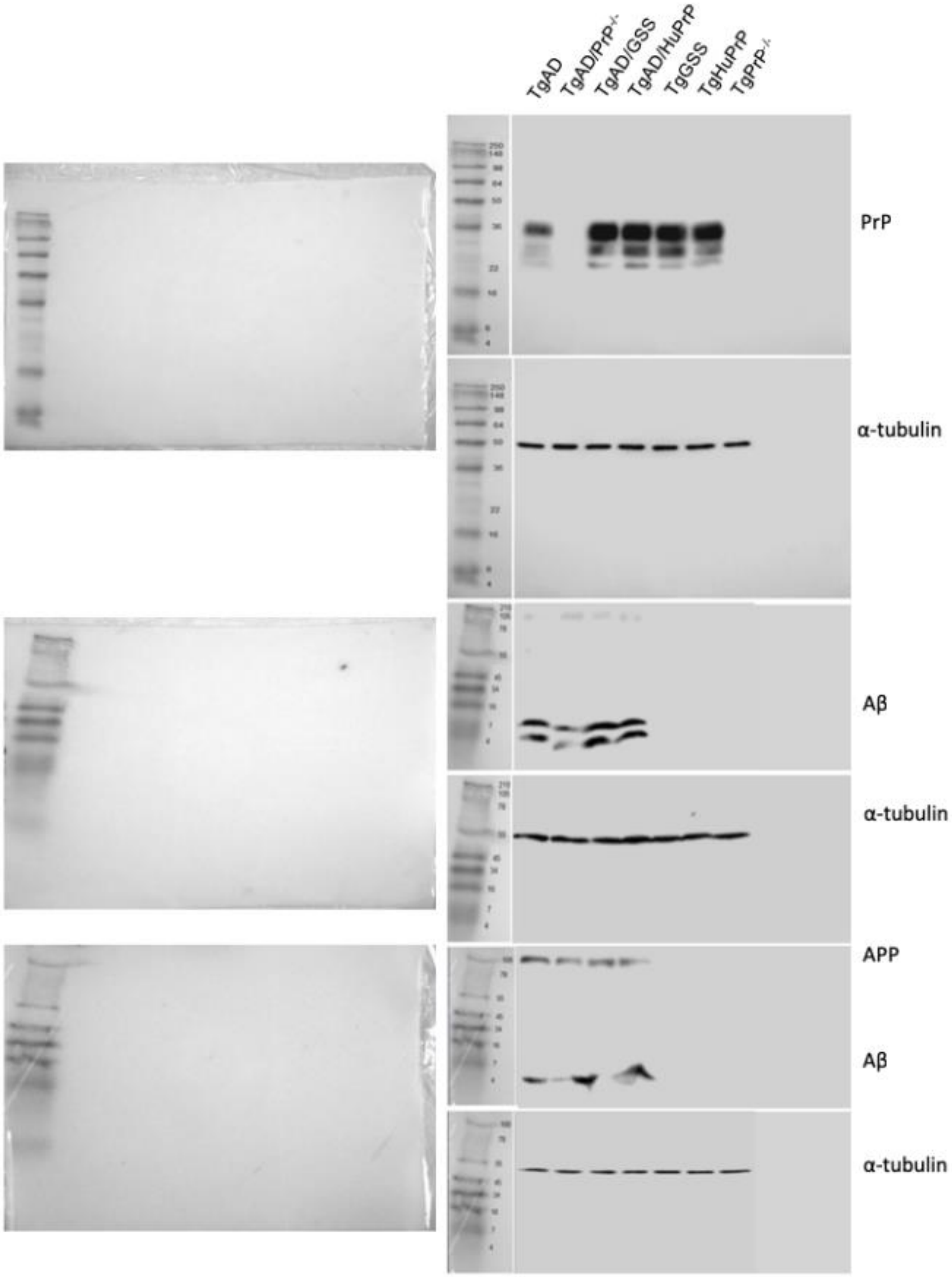

Fig 2. A

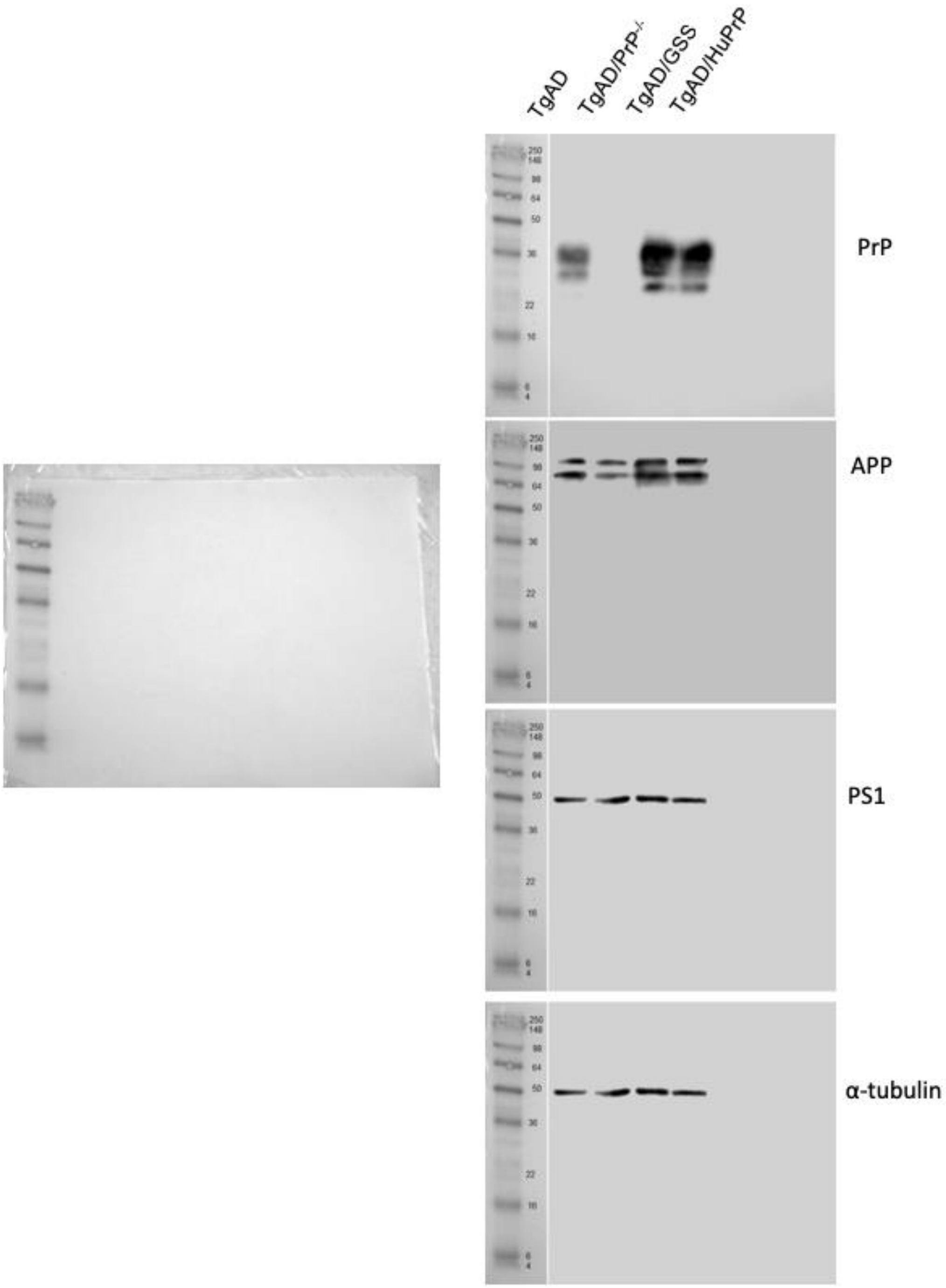

Fig 2. F

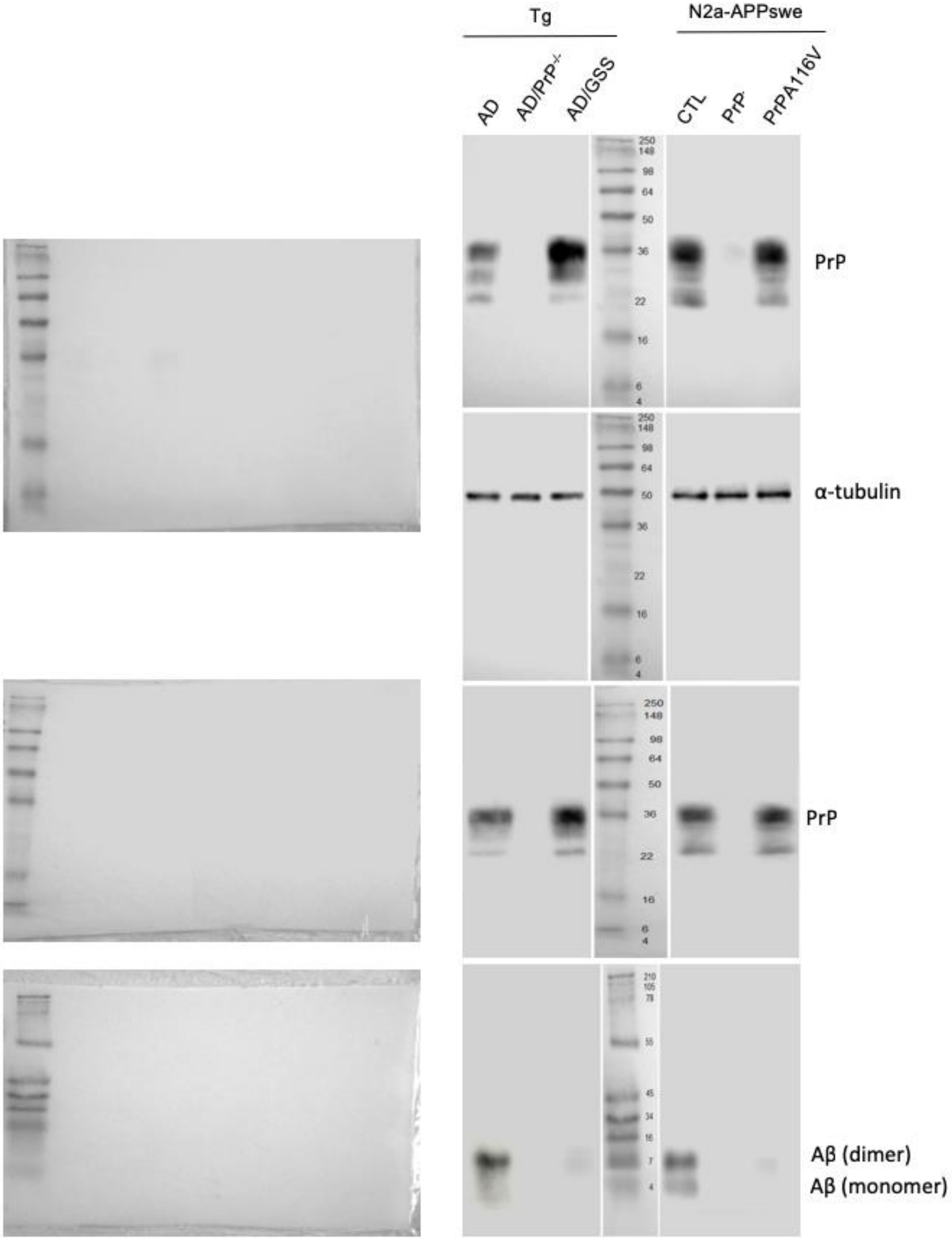

Tg
N2a-APPswe
AD
AD/PrP-/-
AD/GSS
CTL
PrP-
PrPA116V
PrP
α-tubulin
PrP
Aβ (dimer)
Aβ (monomer)

Fig 3. B

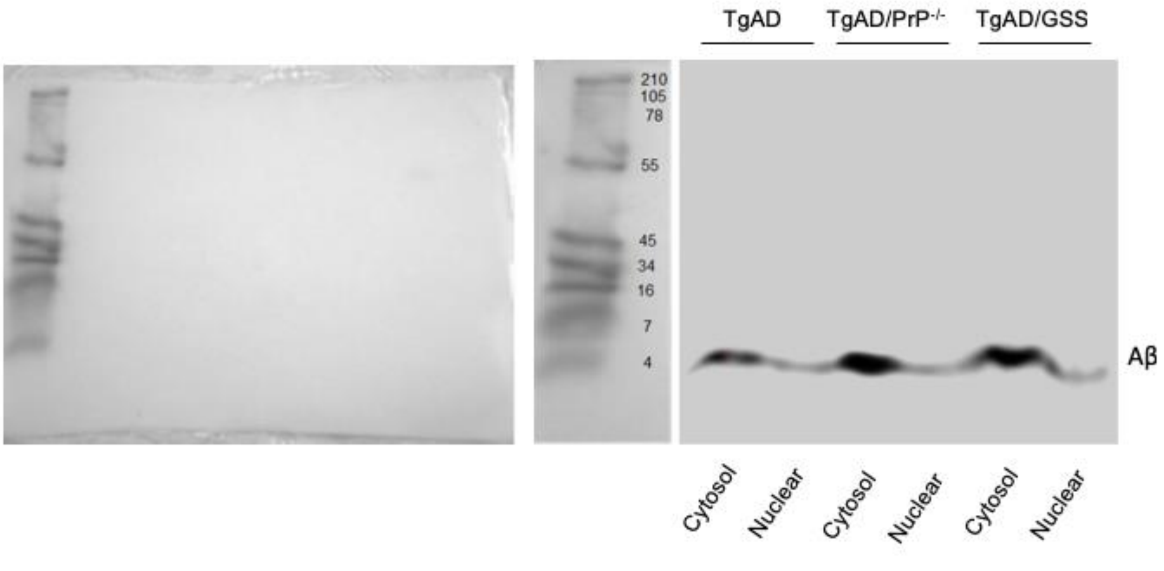
TgAD
TgAD/PrP-/-
TgAD/GSS
210
105
78
55
45
34
16
7
4
Aβ
Cytosol
Nuclear
Cytosol
Nuclear
Cytosol
Nuclear

Fig 4. E

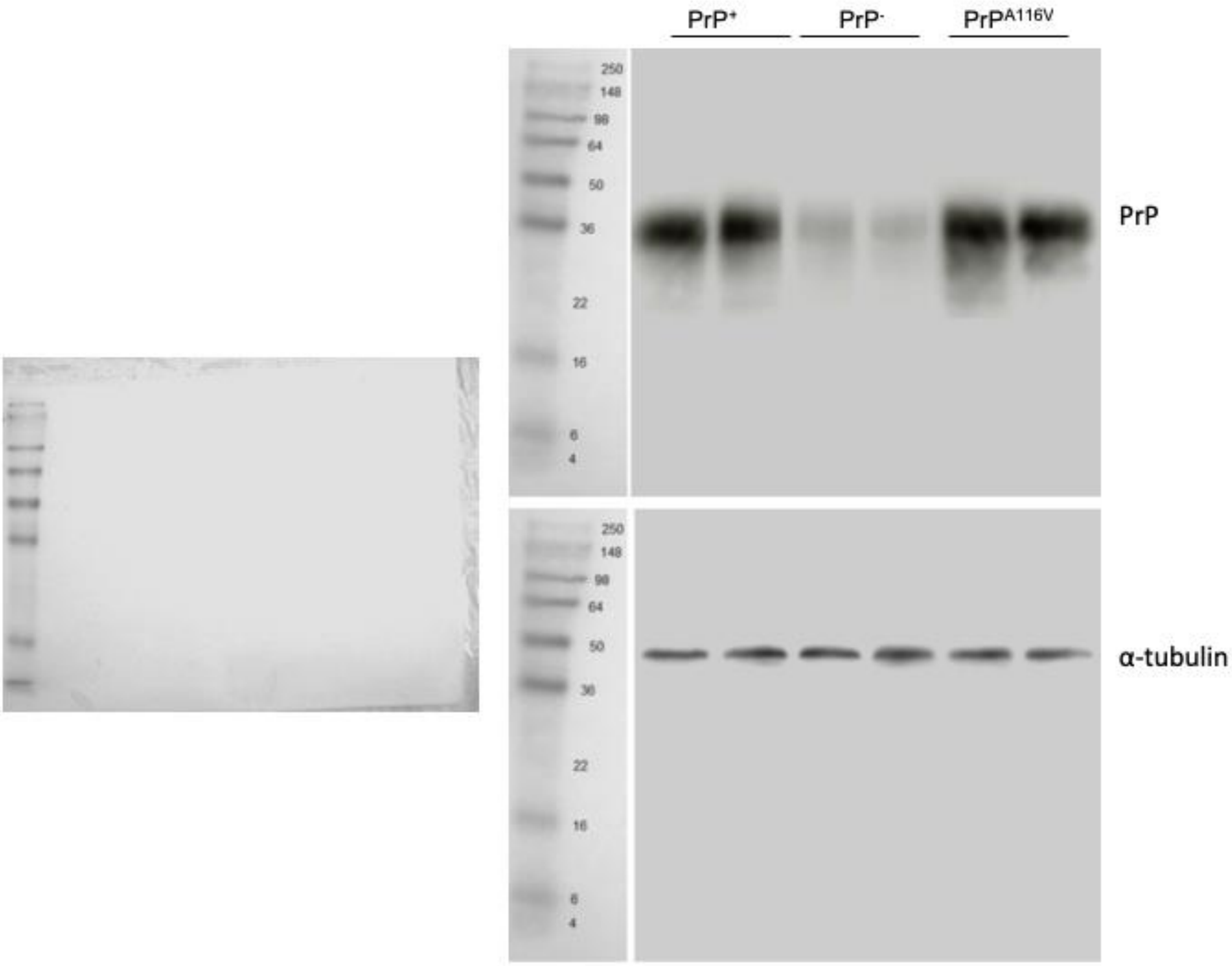

Fig 6. A

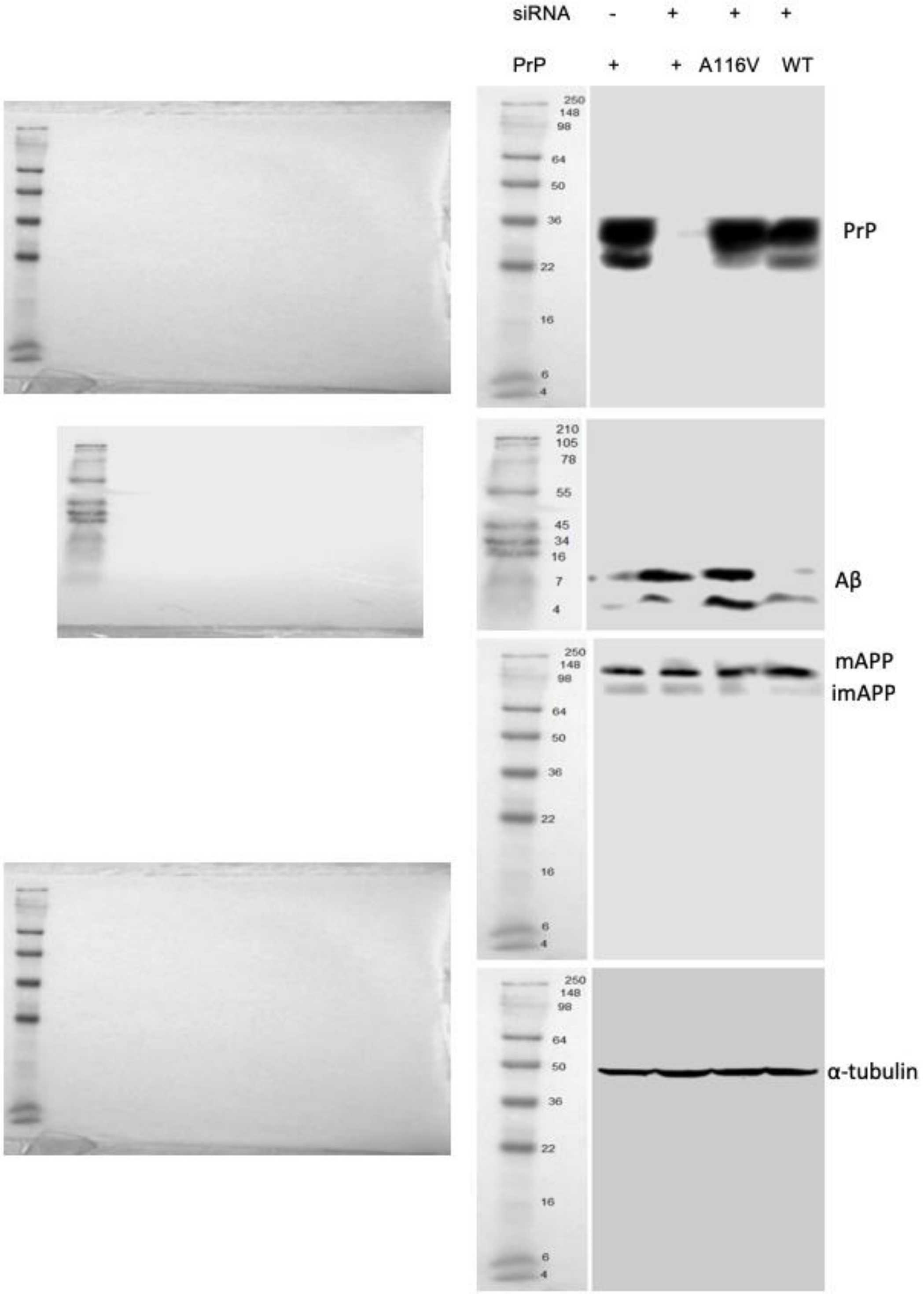

siRNA - + + +
PrP + + A116V WT
250
148
98
64
50
36
22
16
6
4
PrP
210
105
78
55
45
34
16
7
4
Aβ
mAPP
imAPP
α-tubulin

Fig 7. B

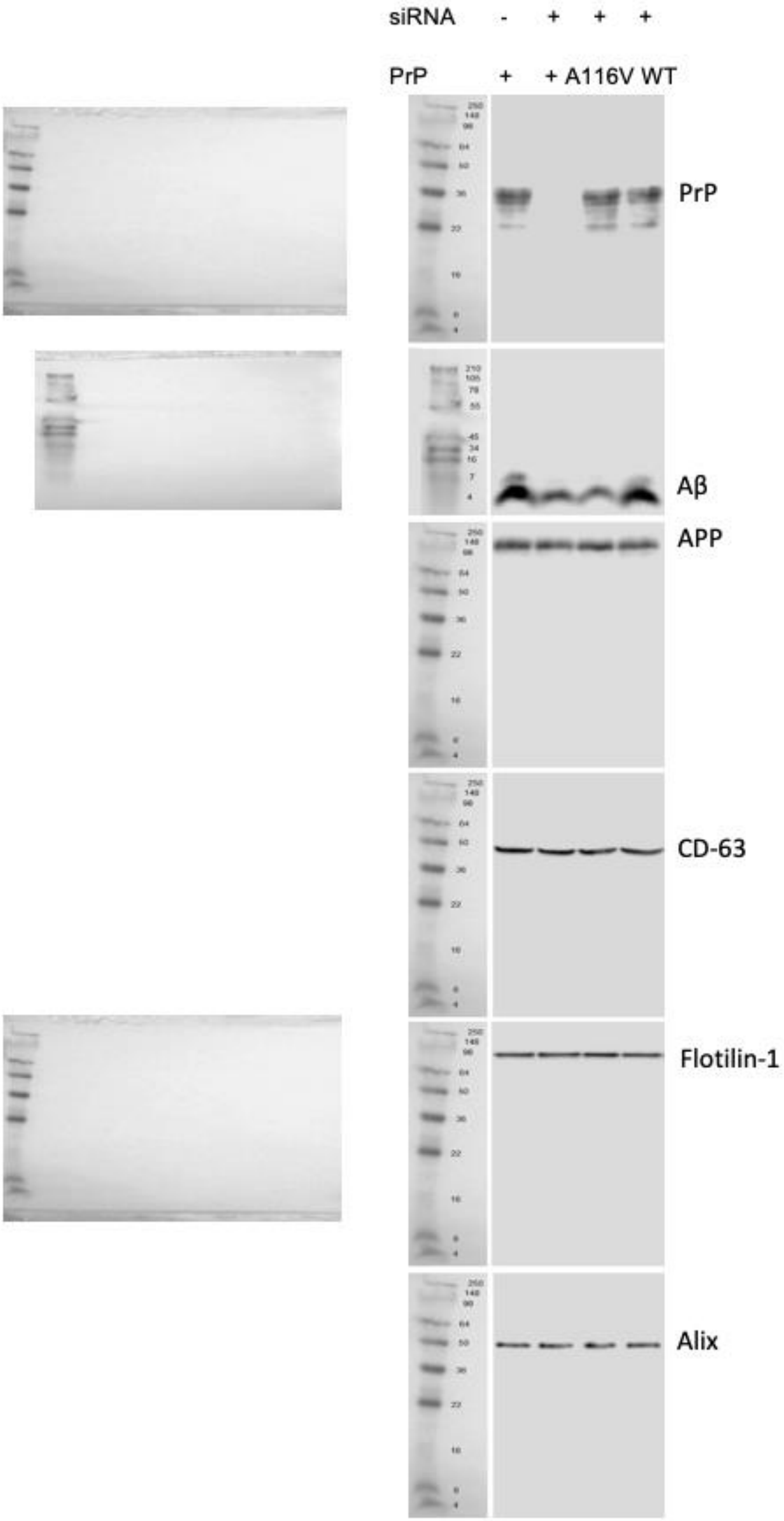

Fig 7. C
